# Temperature-Dependent Network Modules of Soil Methanogenic Bacterial and Archaeal Communities

**DOI:** 10.3389/fmicb.2019.00496

**Published:** 2019-03-12

**Authors:** Pengfei Liu, Melanie Klose, Ralf Conrad

**Affiliations:** Max Planck Institute for Terrestrial Microbiology, Marburg, Germany

**Keywords:** temperature shifts, methanogenesis pathway, methanogenic community, paddy soil, desert soil, functional redundancy

## Abstract

Temperature is an important factor regulating the production of the greenhouse gas CH_4_. Structure and function of the methanogenic microbial communities are often drastically different upon incubation at 45°C versus 25°C or 35°C, but are also different in different soils. However, the extent of taxonomic redundancy within each functional group and the existence of different temperature-dependent microbial community network modules are unknown. Therefore, we investigated paddy soils from Italy and the Philippines and a desert soil from Utah (United States), which all expressed CH_4_ production upon flooding and exhibited structural and functional differences upon incubation at three different temperatures. We continued incubation of the pre-incubated soils (Liu et al., 2018) by changing the temperature in a factorial manner. We determined composition, abundance and function of the methanogenic archaeal and bacterial communities using HiSeq Illumina sequencing, qPCR and analysis of activity and stable isotope fractionation, respectively. Heatmap analysis of operational taxonomic units (OTU) from the different incubations gave detailed insights into the community structures and their putative functions. Network analysis showed that the microbial communities in the different soils were all organized within modules distinct for the three incubation temperatures. The diversity of Bacteria and Archaea was always lower at 45°C than at 25 or 35°C. A shift from 45°C to lower temperatures did not recover archaeal diversity, but nevertheless resulted in the establishment of structures and functions that were largely typical for soil at moderate temperatures. At 25 and 35°C and after shifting to one of these temperatures, CH_4_ was always produced by a combination of acetoclastic and hydrogenotrophic methanogenesis being consistent with the presence of acetoclastic (*Methanosarcinaceae*, *Methanotrichaceae*) and hydrogenotrophic (*Methanobacteriales*, *Methanocellales*, *Methanosarcinaceae*) methanogens. At 45°C, however, or after shifting from moderate temperatures to 45°C, only the Philippines soil maintained such combination, while the other soils were devoid of acetoclastic methanogens and consumed acetate instead by syntrophic acetate oxidation coupled to hydrogenotrophic methanogenesis. Syntrophic acetate oxidation was apparently achieved by *Thermoanaerobacteraceae*, which were especially abundant in Italian paddy soil and Utah desert soil when incubated at 45°C. Other bacterial taxa were also differently abundant at 45°C versus moderate temperatures, as seen by the formation of specific network modules. However, the archaeal OTUs with putative function in acetoclastic or hydrogenotrophic methanogenesis as well as the bacterial OTUs were usually not identical across the different soils and incubation conditions, and if they were, they suggested the existence of mesophilic and thermophilic ecotypes within the same OTUs. Overall, methanogenic function was determined by the bacterial and/or archaeal community structures, which in turn were to quite some extent determined by the incubation temperature, albeit largely individually in each soil. There was quite some functional redundancy as seen by different taxonomic community structures in the different soils and at the different temperatures.

## Introduction

Methane production is achieved by anaerobic degradation of organic matter involving a complex microbial community consisting of Bacteria and Archaea. Bacteria (and perhaps some Archaea) hydrolyze polymers (e.g., cellulose); ferment the monomers to short chain fatty acids, alcohols, CO_2_ and H_2_, or ferment them to acetate as sole product; and convert the primary fermentation products to acetate, H_2_ and CO_2_. Methanogenic archaea then convert either acetate (acetoclastic methanogenesis) or H_2_/CO_2_ (hydrogenotrophic methanogenesis) to CH_4_ (Zinder, 1993; Schink and Stams, 2013). Methane can also be produced from methyl groups such as methanol, trimethylamine, dimethylsulfide etc., if these compounds occur in the environment (Zinder, 1993). In anoxic soils, CH_4_ is usually produced by hydrogenotrophic and acetoclastic methanogenesis. The two pathways can be distinguished, since CH_4_ produced from CO_2_ reduction is much more depleted in ^13^C than that from acetate cleavage (Whiticar, 1999; Conrad, 2005). Soils usually contain a rather high diversity of Archaea, including methanogens such as the potentially acetoclastic families *Methanosarcinaceae* and *Methanotrichaceae* [‘*Methanosaetaceae*’(Oren, 2014)] as well as the hydrogenotrophic orders *Methanocellales*, *Methanomicrobiales*, and *Methanobacteriales* (Conrad, 2007). The complexity of Bacteria is even larger (Asakawa and Kimura, 2008; Rui et al., 2009; Wegner and Liesack, 2016). Both domains (Archaea and Bacteria) include a large number of taxa of which isolates are lacking and their potential physiology is little known or completely unknown. The extent of taxonomic redundancy within each functional group (e.g., acetoclastic methanogenesis) is also not well known. Such redundancy may be promoted by differentiation in response to environmental factors other than metabolic resources (Louca et al., 2018). Temperature is such an environmental factor and thus, we used it in the present study to affect microbial function and taxonomic structure.

Temperature is a universal regulator of microbial activity (Wiegel, 1990), and also greatly affects CH_4_ production (Yvon-Durocher et al., 2014). Microbial community responses to increasing temperatures can be quite complex (Roussel et al., 2015). Temperature not only regulates each individual process by affecting the underlying thermodynamics and kinetics, but also affects population dynamics and the structuring of the entire microbial community (Conrad, 2008). Rice field soils, for example, are able to produce CH_4_ over a wide temperature range up to about 55°C (Yao and Conrad, 2000; Fey et al., 2001). However, the structure and function of the methanogenic microbial community was found to change drastically within a temperature interval of about 42–46°C (Conrad et al., 2009). Whereas a mesophilic methanogenic community exists below this interval, above it is replaced by a thermophilic one. This change often coincides with a change in the methanogenic pathway from a mixture of acetoclastic plus hydrogenotrophic to exclusively hydrogenotrophic methanogenesis, and by the replacement of acetoclastic methanogenesis by syntrophic acetate oxidation that is coupled to hydrogenotrophic methanogenesis (Conrad et al., 2009; Liu and Conrad, 2010; Rui et al., 2011). Recently, metatranscriptomics and co-occurrence network analysis revealed that the microbial community of Italian rice field soil consisted of modules characterized by the functional activities of polymer hydrolysis, syntrophic oxidation of key intermediates, and methanogenesis (Peng et al., 2018). Both mesophilic (30°C) and moderate thermophilic (45°C) temperatures had a differential effect on all the functional activities (Peng et al., 2018).

In a previous study, we found different methanogenic pathways, depending on whether the soils did or did not contain thermophilic acetoclastic methanogens (Liu et al., 2018). In fact, many different thermophilic methanogens exist in anoxic soils from various regions (Wu et al., 2006), and only few soils have so far been tested with respect to temperature effects on methanogenic microbial communities (Conrad et al., 2009; Rui et al., 2011; Lu et al., 2015; Liu et al., 2018). Therefore, we asked whether the methanogenic microbial communities in soil will always be organized in co-occurrence network modules, which are distinct for different temperatures. Consequently, the microcosms of the three soils incubated for ∼250 days and analyzed in our previous study (Liu et al., 2018) were now considered as a pre-incubation in this study, where their incubation temperature was changed in a factorial manner. They were incubated for a further 30–50 days and investigated for the composition of bacterial and archaeal operational taxonomic units (OTUs) of the resulting communities. The temperature shifts were applied to evaluate to which extent the microbial communities were able to respond by changing composition, in particular to see to which extent a mesophilic community would regenerate after exposure to elevated temperatures and vice versa.

The diversity of the methanogenic archaeal and bacterial communities seems to be lower under thermophilic than mesophilic conditions. Nevertheless, mesophilic communities and functions can be recovered from thermophilic ones when the temperature is decreased below the temperature interval of about 42–46°C (Conrad et al., 2009; Noll et al., 2010), especially if the soil is inoculated with a mesophilic community. Structure and function of the methanogenic communities apparently change in parallel. However, changes in communities have so far only been recorded by molecular fingerprint analysis, which addresses the composition of the communities only coarsely. This is especially true for bacterial communities (Noll et al., 2010). With the availability of deep sequencing techniques (e.g., Illumina) such methodological limitations are now released. Therefore, and because we recently discovered quite different structures of mesophilic and thermophilic methanogenic communities in different soils (Liu et al., 2018), we decided to investigate how these structures and their functions change when incubation temperatures are shifted between mesophilic and thermophilic conditions.

The overall objectives of our study were to unravel the taxonomic redundancy of functional microbial groups, in particular syntrophic acetate oxidation and acetoclastic and hydrogenotrophic methanogenesis, after exposure to mesophilic (25 and 35°C) and thermophilic (45°C) temperatures, and to find out whether these communities were organized in co-occurrence networks. We first hypothesized that function (pathway of CH_4_ production in particular) at different temperatures would be determined by the existence or non-existence of specific functional microbial groups. We secondly hypothesized that these functional groups may form different taxonomic community structures depending on soil and temperature. Thirdly, we asked whether taxonomic diversity and putative functional redundancy are affected by temperature and by shifts between these temperatures.

We used two different paddy soils (from Italy and from the Philippines) and one desert soil (from Utah, United States), which all produced CH_4_ in anoxic soil slurries. After pre-incubation at mesophilic (25 and 35°C) or thermophilic (45°C) conditions, temperatures were changed in a factorial manner. The methanogenic pathways were determined by stable isotope fractionation and the microbial community structures by the taxonomic composition and abundance of Bacteria and Archaea.

## Materials and Methods

### Soil Sampling, Soil Incubation and Chemical Analyses

The same soils from three different climate zones were used as in our previous study (Liu et al., 2018), including two paddy soils from Italian Rice Research Institute in Vercelli (collected in 2013) and the International Rice Research Institute (IRRI) in Los Baños, Philippines (collected in 2012), respectively, and one desert soil from a natural site in Utah, United States (collected in 2009). The physicochemical properties of each soil were measured by standard methods (Fu et al., 2018). The same batch of desert soil had been used in a previous study (Angel et al., 2012). The incubations were set up in 2015 and continued into the year 2016.

Anoxic soil incubations were separated into two phases. During pre-incubation, soil slurries were prepared by mixing 50 g dry soil with 50 ml of deionized, sterile, anoxic water and 100 mg cellulose in 150-ml bottles. The bottles were sealed with rubber stoppers and flushed with N_2_, and then incubated at three different temperatures (each with three replicates): 25, 35, and 45°C. Treatments were designated as I25, I35, and I45 for Italian paddy soil, P25, P35, and P45 for the Philippines paddy soil and U25, U35, and U45 for Utah soil, respectively (Supplementary Figure S1). After about 250 days incubation, aliquots of soil slurries were collected and stored frozen at −20°C for later molecular analysis. Part of the data from the pre-incubation have been reported in our previous paper (Liu et al., 2018), i.e., production rates and isotopic compositions of CO_2_ and CH_4_; relative abundance of the major bacterial and archaeal phyla; copy numbers of bacterial and archaeal 16S rRNA genes; concentrations of volatile fatty acid. If required for comparison, they are included in the present manuscript as mentioned in the legends of tables and figures. However, the composition of the microbial communities in terms of OTUs has not yet been reported and is subject of the present study.

The remaining soil slurries from the three triplicates at the same temperature were mixed and dispensed into 26-mL tubes, with 10 g slurry per tube. The soil slurries were kept stirred by a high speed magnetic mixer to avoid particles settling during the dispensing of preincubated soil. In addition, the soil slurries were amended with 10 mg of cellulose as additional carbon source. The tubes were closed with black rubber stoppers, flushed with N_2_, pressurized to 0.5 bar overpressure. The soil slurries were either continuously incubated under the same temperature as during pre-incubation or switched to one of the other two temperatures (Supplementary Figure S1). In total, nine treatments (each with three replicates) were established for each soil. For Italian paddy soil, treatments were designated as I25e25, I25e35, I25e45, and I35e25, I35e35, I35e45, and I45e25, I45e35, I45e45. For the Philippines paddy soil, treatments were designated as P25e25, P25e35, P25e45, and P35e25, P35e35, P35e45, and P45e25, P45e35, P45e45. For Utah desert soil, treatments were designated as U25e25, U25e35, U25e45, and U35e25, U35e35, U35e45, and U45e25, U45e35, U45e45 (Supplementary Figure S1). Soil slurries were incubated at the respective temperatures for 30–50 days. During the incubation, accumulation of CH_4_ and CO_2_ in the headspace was measured using a gas chromatography (GC) equipped with methanizer and flame ionization detector as previously described (Yao et al., 1999). The δ^13^C of CH_4_ and CO_2_ was measured by GC combustion isotope ratio mass spectrometry (GC-C-IRMS) (Conrad et al., 2012). The apparent isotopic fractionation factor for conversion of CO_2_ to CH_4_ was determined by α_app_ = (δ^13^CO_2_ + 10^3^)/(δ^13^CH_4_ + 10^3^) (Fey et al., 2004). Volatile fatty acids were measured by high-performance liquid chromatography (HPLC) (Liu and Conrad, 2010). Soil slurries of each treatment were collected at the end of incubation and stored frozen at −20°C for later analysis of volatile fatty acids and for molecular analysis.

### DNA Extraction, qPCR, Illumina Library Preparation and Sequencing

The same protocol was used for DNA extraction and quantification of archaeal and bacterial 16S rRNA genes by qPCR as in our previous study (Liu et al., 2018).

The Illumina libraries were prepared as in our previous study (Liu et al., 2018) using primers 515F and 806R targeting the V4 region of the 16S rRNA gene (approximately 250 nucleotides) for both Archaea and Bacteria (Bates et al., 2011). Library 1 included all samples from the end of the pre-incubation (Liu et al., 2018). Libraries 2–4 included samples of Italian, the Philippines and Utah soils, respectively, at the end of incubation at different temperatures. All libraries were sequenced on an ILLUMINA HISEQ 2000 system using 2 × 250 cycle combination mode by Max Planck-Genome-Centre (Cologne, Germany). Sequence data sets were stored in the same project as our previous study (Liu et al., 2018) with accession number SRP133538.

### Bioinformatic Analyses

Similar to our previous study (Liu et al., 2018), sequence processing was mainly based on the UPARSE pipeline (Edgar, 2013) and additionally included tools from the programs QIIME 1.9.0 (Caporaso et al., 2010), CUTADAPT 1.9.1 (Martin, 2011), and USEARCH v8.0.1623 (Edgar, 2010). In brief, the adaptor sequence ahead of the barcoded forward primer and reverse primers (Supplementary Table S1) were trimmed with CUTADAPT. Un-trimmed sequences were discarded. Paired-end reads were then merged and demultiplexed using UPARSE python scripts^1^. After quality control, *de novo* chimera filtering and singleton filtering, species level OTUs for 16S rRNA genes were obtained at 97% sequence identity. Taxonomic identities of 16S rRNA gene OTUs were assigned with the Ribosomal Database Project (RDP) Classifier against the SILVA 128 SSU Ref database (Pruesse et al., 2007) at a confidence level of 80% (Wang et al., 2007). OTUs belonging to Bacteria and Archaea were separated according to their taxonomic identities. For alpha-diversity analyses, the OTU tables with singletons were subsampled using the rrarefy function of the vegan package (version 2.4-4) (Oksanen et al., 2007) within R^2^ based on the lowest number of sequences available from each samples, i.e., 281947 for bacterial- and 10162 for archaeal-16S rRNA genes. This procedure standardizes the measures needed for comparison. Rarefied OTU tables were then processed within packages vegan, entropart (version 1.4-7) and phyloseq (version 1.19.1) (McMurdie and Holmes, 2013) in R. Bray–Curtis distance matrix was calculated based on OTUs tables without singletons, non-metric multidimensional scaling analysis (NMDS) was then carried out using the ordination function within R package phyloseq (McMurdie and Holmes, 2013). Differences in population structure between treatments within each types of soils were analyzed using the statistic ANOSIM (based on Bray–Curtis dissimilarities, permutations = 999) in R.

Heatmaps were constructed with the pheatmap (pretty heatmaps version 1.0.8) and vegan packages using OTUs that explained most of the differences between samples. Such OTUs were identified by principal components analysis (PCA) of the Hellinger transformed data using the prcomp function in vegan. Twenty five archaeal OTUs and 50 bacterial OTUs explaining most of the differences between samples were defined as the OTUs contributing the largest absolute loadings in the first and second dimensions of the PCA (Hernandez et al., 2017), obtained from the rotation output file. To construct heatmaps for Archaea, a total of 35, 39, and 32 unique OTUs were obtained for Italian, the Philippines and Utah soil, respectively (since some of the OTUs were selected from more than one PCA axes). To construct heatmaps for Bacteria, a total of 88, 86, and 85 unique OTUs were obtained for bacterial 16S rRNA genes for Italian, the Philippines and Utah soil, respectively. The OTU abundances were converted to percentage of sequences from each sample, and Hellinger distances were calculated as described above. Hierarchical clustering of the distance matrix was carried out with the “complete” method using hclust function.

### Co-occurrence Network Analysis

Three co-occurrence networks based on the abundance of 16S rRNA gene OTUs (including both bacterial and archaeal OTUs) were constructed for Italian, the Philippines and Utah soils, respectively, by following the pipelines developed by Williams et al. (2014). Generally, the OTU table without singletons was first rarefied to the smallest sample depth for all three soils (here 281947 reads for bacterial 16S rRNA gene and 10162 reads for archaeal 16S rRNA gene). OTUs with less than 0.1% or 1% maximum relative abundances for Bacteria and Archaea, respectively, were filtered out. OTUs from Bacteria and Archaea were combined and Spearman’s rank correlations between selected OTUs were calculated based on absolute reads abundance. Pairs with Spearman’s correlation coefficient 0.8 ≤ ρ ≤ 0.9 and FDR corrected *p*-value < 0.01 were used for co-occurrence network construction. Networks were constructed with the robust correlations as weighted edges using Gephi software^3^. The network layout was generated using the force-based algorithm Fruchterman–Reingold (Fruchterman and Reingold, 1991). Modules within network were computed using the multilevel modularity optimization algorithm (Blondel et al., 2008).

### Statistical Analyses

For qPCR data, the means ± standard deviation (SD) from all replicates were calculated. Gene copy numbers were log-transformed to satisfy the normality assumptions, and the analysis of variance was performed to test significant differences between treatments using the DUNCAN test within agricolae package (version 1.2-8) (Mendiburu and Simon, 2015) in R. For the α diversity index, analysis of variance was performed to test significant differences between treatments using the Kruskal–Wallis test within agricolae package.

## Results

### Functional Process Data

**FIGURE 1:**
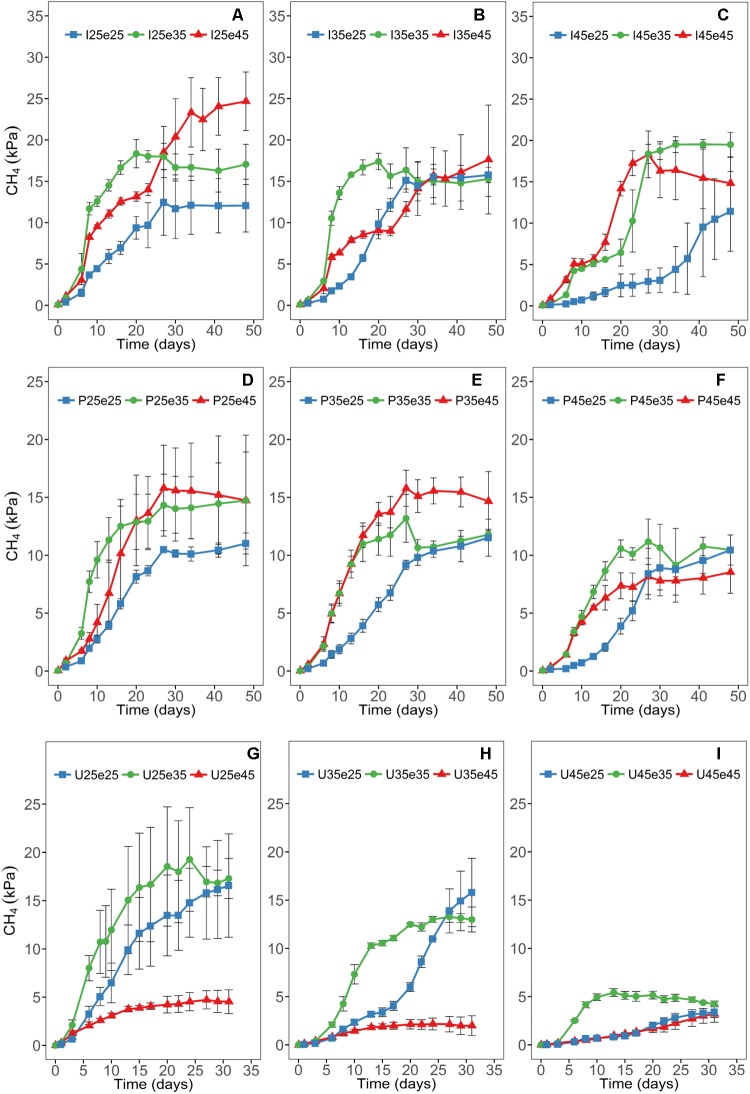
Time courses of accumulation of CH_4_ at different temperatures in Italian **(A–C)** and the Philippines **(D–F)** paddy soils and in Utah desert soil **(G–I)**; data are means ± SD (*n* = 3). Panels are arranged for each soil according to the temperatures of the pre-incubation at 25, 35, or 45°C. See main text and Supplementary Figure S1 for the description of each treatment.

Patterns of CH_4_ production (Figure 1) and CO_2_ production (Supplementary Figure S2) were dependent on the temperature during pre-incubation and were different at 25, 35, and 45°C (Figure 1). Production rates of CO_2_ tended to increase when the temperature was increased after pre-incubation and vice versa (Supplementary Figure S2). Also CH_4_ production increased when shifting from 25°C during pre-incubation to 35°C (Figure 1). Shifting to 45°C also increased CH_4_ production in the paddy soils (Italy, the Philippines), but decreased them in the Utah desert soil (Figure 1). Shifting from 35 to 25°C generally resulted in a lag phase of several days and in decreased CH_4_ production (Figure 1). Shifting from 45°C during pre-incubation to lower temperatures resulted in similar rates at 35°C, but significantly decreased the rates at 25°C (Figure 1). However, this pattern was only observed in the two paddy soils (Italy, the Philippines), while in the Utah desert soil CH_4_ production at 25 and 45°C became very low (Figure 1).

**FIGURE 2:**
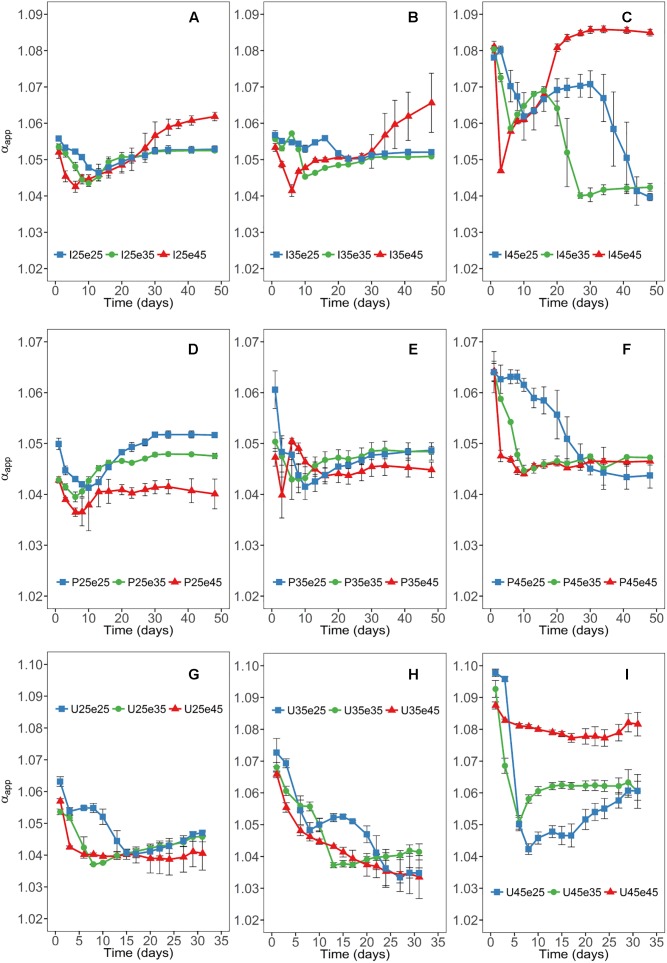
Time courses of apparent isotope fractionation factors (α_app_) at different temperatures in Italian **(A–C)** and the Philippines **(D–F)** paddy soils and in Utah desert soil **(G–I)**; data are means ± SD (*n* = 3). Panels are arranged for each soil according to the temperatures of the pre-incubation at 25, 35, or 45°C. See Section “Materials and Methods” in the main text and Supplementary Figure S1 for the description of each treatment.

High values of α_app_ (about 1.08) are characteristic for CH_4_ production exclusively from CO_2_ and low values (about 1.04) of α_app_ are characteristic for CH_4_ production from both CO_2_ and acetate (Fey et al., 2004). Continuous incubation at 45°C resulted in the establishment of high α_app_ values in Italian paddy soil and Utah desert soil indicating that CH_4_ was mainly (or exclusively) produced from H_2_/CO_2_ (Figure 2). In the Philippines paddy soil, by contrast, α_app_ values eventually became relatively low indicating that CH_4_ was produced by both acetoclastic and hydrogenotrophic methanogenesis (Figure 2). This happened in the Philippines soil at all temperatures irrespectively of pre-incubation (Figure 2). Relatively low α_app_ values also established in all the other soils at 25 and 35°C irrespectively of the temperature of pre-incubation. However, increase of the pre-incubation temperature of Italian paddy soil (but not of Utah desert soil) to 45°C resulted in an increasing trend of α_app_ (Figure 2).

We also determined the concentrations of volatile fatty acids (VFA) at the end of incubation (Supplementary Table S2). In the paddy soils, acetate, propionate and formate were detected, the concentrations being below 1 mM. Capronate was only detected in I25e45. In the Utah desert soil, however, also lactate, butyrate, valerate, iso-valerate, and capronate were frequently detected (Supplementary Table S2). Concentrations of acetate were in Utah desert soil especially high (2–6 mM), and were exceptionally high if temperature was decreased from 45°C during pre-incubation to 25°C or 35°C (reaching >70 mM) (Supplementary Table S2). The accumulation of VFA is consistent with the very low rates of CH_4_ production after pre-incubation of Utah soil at 45°C (Figure 1). The pH values were generally around neutrality. However, in Utah soil after pre-incubation at 45°C values were between pH 6.0 and 6.5.

### Quantification of Archaeal and Bacterial 16S rRNA Genes

**FIGURE 3:**
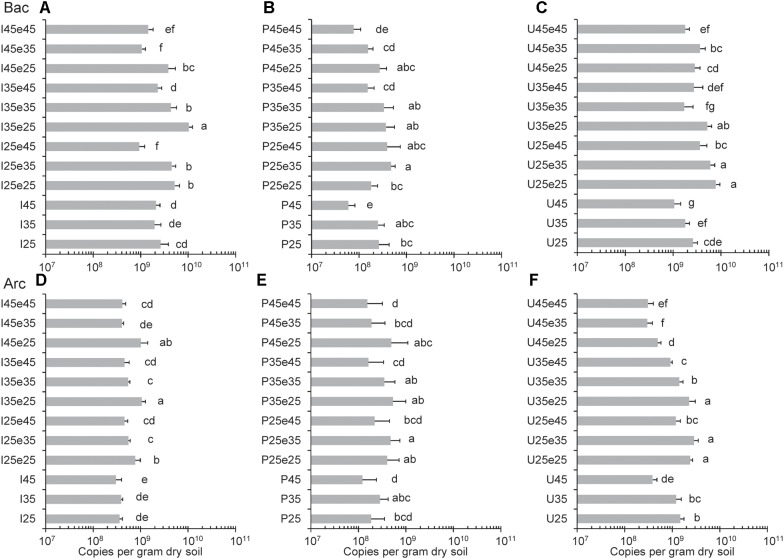
Copy numbers of **(A,B,C)** Bacterial and **(D,E,F)** Archaeal 16S rRNA genes in **(A,D)** Italian paddy soil, **(B,E)** the Philippines paddy soil and **(C,F)** Utah desert soil; data are means ± SD, with *n* = 3. Different letters next to the data indicate significant difference (*p* < 0.05) between copy numbers. See Section “Materials and Methods” in the main text and Supplementary Figure S1 for the description of each treatment. Data from the pre-incubation (I25, 35, 45, P25, 35, 45, and U25, 35, 45) were reported in our previous publication (Liu et al., 2018).

Copy numbers of archaeal and bacterial 16S rRNA genes were quantified to assess the absolute abundances (gene copies) of Archaea and Bacteria (Figure 3). Copy numbers of archaeal 16S rRNA genes were generally between 1 × 10^8^ and 5 × 10^9^ copies per gram dry soil across all the samples. Copy numbers of bacterial 16S rRNA genes were nearly one order of magnitude higher than archaeal 16S rRNA genes in Italian and Utah soil and they were similar in the Philippines soil (Figure 3). Copy numbers of both archaeal and bacterial 16S rRNA genes were usually slightly higher (often significantly, *p* < 0.05) in samples incubated at moderate temperatures than at 45°C. In Utah desert soil, copy numbers of archaeal genes stayed relatively low once soil had been incubated at 45°C. Another interesting feature was that the copy numbers (both Bacteria and Archaea) increased from the end of pre-incubation to the end of the subsequent incubation indicating microbial growth (Figure 3).

### Overall Microbial Diversity and Composition of Microbial Communities

**FIGURE 4:**
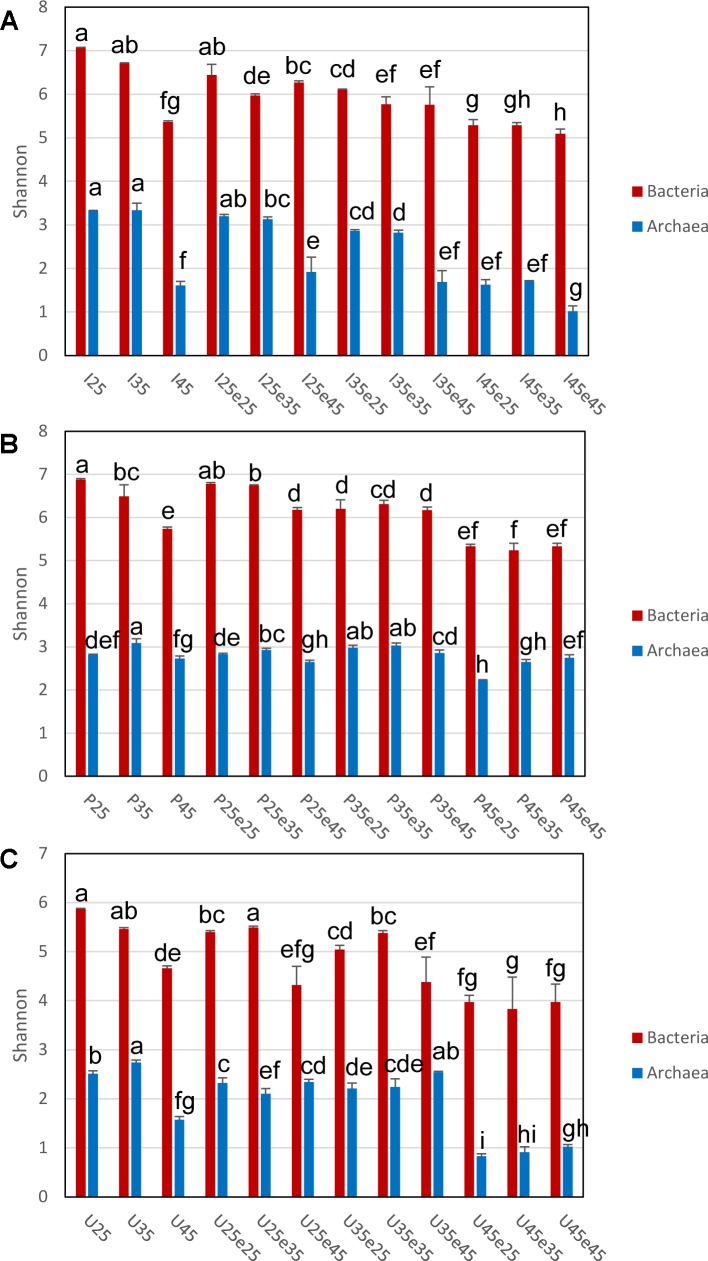
Shannon indices of Bacterial and Archaeal 16S rRNA genes in **(A)** Italian paddy soil, **(B)** the Philippines paddy soil and **(C)** Utah desert soil; data are means ± SD, with *n* = 3. Different letters above the data indicate significant difference (*p* < 0.05) between different treatments, with Bacterial and Archaeal 16S rRNA genes being tested separately. See Section “Materials and Methods” in the main text and Supplementary Figure S1 for the description of each treatment. Other alpha diversity indices (e.g., species richness) are provided in Supplementary Tables S3, S4.

The composition of the microbial communities in the different treatments of the three soils was assessed by HiSeq sequencing, resulting in an average of 12–80 thousands and 300–500 thousands reads for Archaea and Bacteria, respectively (Supplementary Tables S3, S4). The average numbers of OTUs obtained by similarity-based clustering of the sequences (3% sequence dissimilarity and without singletons) were about 40–200 and 2500–9200 for Archaea and Bacteria, respectively. Current sequencing depth covered almost the entire archaeal and bacterial diversity in all three soils, although some rare taxa were probably missed (Supplementary Tables S3, S4). For both Archaea and Bacteria, pre-incubation at 45°C or increase of the temperature to 45°C often significantly decreased diversity as described by species richness (Chao1 estimators), Shannon and Fisher indices and evenness (J). The decrease was most pronounced in Italian paddy soil and Utah desert soil, but was less obvious in the Philippines paddy soil (as exemplified by the Shannon index in Figure 4). By contrast, decrease of the temperature from 45°C during pre-incubation to 25°C or 35°C did not result in significant increase of diversity. Apparently, diversity was not recovered once it had been lost.

At the end of the pre-incubation at 25, 35, and 45°C the archaeal communities in the different soils were different, and again changed with continuing incubation (Supplementary Figure S3). The Italian paddy soil at 25 and 35°C mainly contained *Methanobacteriales*, *Methanocellales*, *Methanosarcinaceae*, and *Methanotrichaceae*, but also some *Methanomicrobiales*. In addition there were *Bathyarchaeota*, Soil Crenarchaeota Group (SCG), *Woesarchaeota* and also some *Thermoplasmatales* (Supplementary Figure S3A). This composition was rather well maintained upon continued incubation at the same temperature or upon shift to another moderate temperature. In the Philippines paddy soil, the archaeal communities were qualitatively similar at all the different temperatures (Supplementary Figure S3B). Notably, the community always consisted of all the major archaeal groups, including *Methanobacteriales*, *Methanocellales*, *Methanomicrobiales*, *Methanosarcinaceae*, *Methanotrichaceae*, *Bathyarchaeota*, SCG, *Woesarchaeota*, and *Thermoplasmatales* (Supplementary Figure S3B). Hence, acetoclastic *Methanosarcinaceae* and *Methanotrichaceae* were always present. The archaeal community in Utah desert soil rarely contained acetoclastic *Methanotrichaceae*, and did not contain *Woesearchaeota* (Supplementary Figure S3C). At 45°C Utah desert soil also did not contain any acetoclastic *Methanosarcinaceae*, and did not contain *Bathyarchaeota*. Further incubation at 45°C resulted in increase of *Methanobacteriales* (∼80%) while *Methanocellales* almost disappeared (Supplementary Figure S3C).

The bacterial communities consisted of 18 different taxonomic classes with >2% relative abundance (Supplementary Figure S4). Pre-incubation at 45°C generally resulted in higher relative abundance of *Clostridia* and other *Firmicutes* than pre-incubation at moderate temperatures (Supplementary Figure S4). Decrease of temperature did not reverse this pattern. In addition to the general responses of bacterial compositions, we also investigated the changes of putative syntrophic bacteria including *Thermoanaerobacteraceae*, *Peptococcaceae*, *Syntrophomonadaceae*, and *Helicobacteraceae* (all belonging to the phylum *Clostridia*), as well as *Syntrophobacteraceae*, *Syntrophaceae*, and *Geobacteraceae* (all belonging to the class *Delta-Proteobacteria*) (Supplementary Figure S5). In particular, *Thermoanaerobacteraceae* were interesting, since they represent putative acetate oxidizing syntrophs. In Italian soil, this group always showed a relatively high abundance, when the soil was incubated at 45°C (Supplementary Figure S5A), while in the Philippines soil, the abundance of *Thermoanaerobacteraceae* was generally low (Supplementary Figure S5B).

Non-metric multidimensional scaling (NMDS) analysis of archaeal OTUs showed differences in community compositions between all the different treatments and for all the three soils (Supplementary Figure S6), and many of these differences were significant (see *R*- and *P*-values in Supplementary Figure S6). For Italian soil, the I45e45 treatment formed a unique archaeal cluster. Other samples incubated at 45°C either during pre-incubation or after temperature change clustered together and were separated from samples that were exclusively incubated at moderate temperatures (Supplementary Figure S6A). For Utah and the Philippines soils, samples were mainly separated by the incubation temperature during pre-incubation (Supplementary Figures S6B,C).

Non-metric multidimensional scaling also confirmed that the bacterial communities within each soil were significantly different for the different treatments (see *R*-and *P*-values in Supplementary Figure S7). Similar with Archaea, the Bacteria in the samples were mainly separated by the incubation temperature during pre-incubation, and there was little effect by the temperature change, except when temperature was increased to 45°C (Supplementary Figure S7).

### Heatmap Analysis of Microbial Operational Taxonomic Units

**FIGURE 5:**
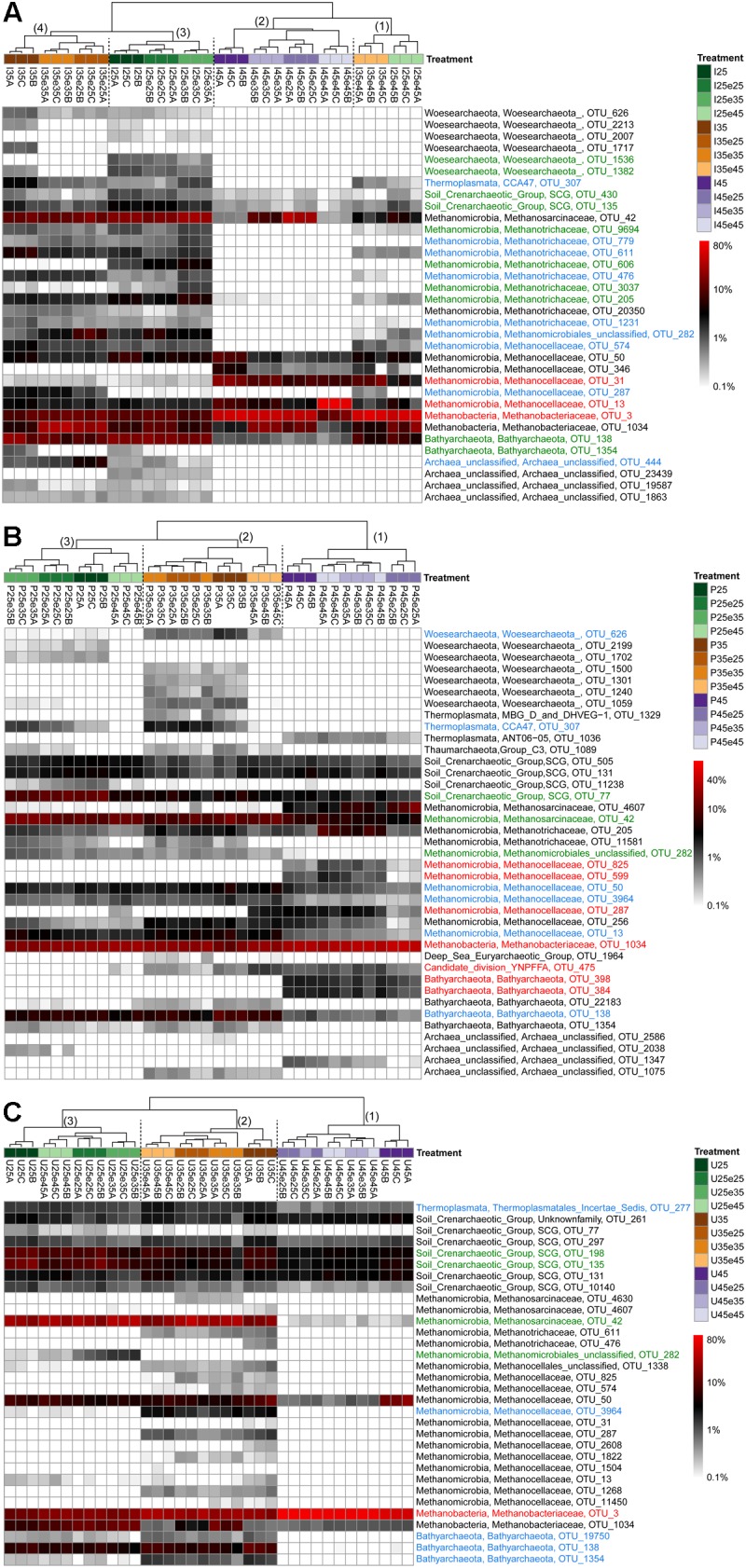
Relative abundances of selected archaeal OTUs that explain most of the differences between treatments in **(A)** Italian paddy soil, **(B)** the Philippines paddy soils, and **(C)** Utah desert soil. Descriptions of OTUs selection, sample clustering and heatmap construction are given in Section “Materials and Methods” in the main text. See Section “Materials and Methods” in the main text and Supplementary Figure S1 for the description of each treatment. The color scale is logarithmic to emphasize rare taxa, with 0.1% being set as the lower bound for logarithmic transformation. Numbers above the dendrogram indicate clusters described in the main text. Names of the OTUs were colored according to the three major modules in the Supplementary Figures S8–S10, i.e., green, blue, and red corresponding to module 25, 35, and 45°C, respectively (see Supplementary Figures S8–S10). OTUs in black were not in the network.

**FIGURE 6:**
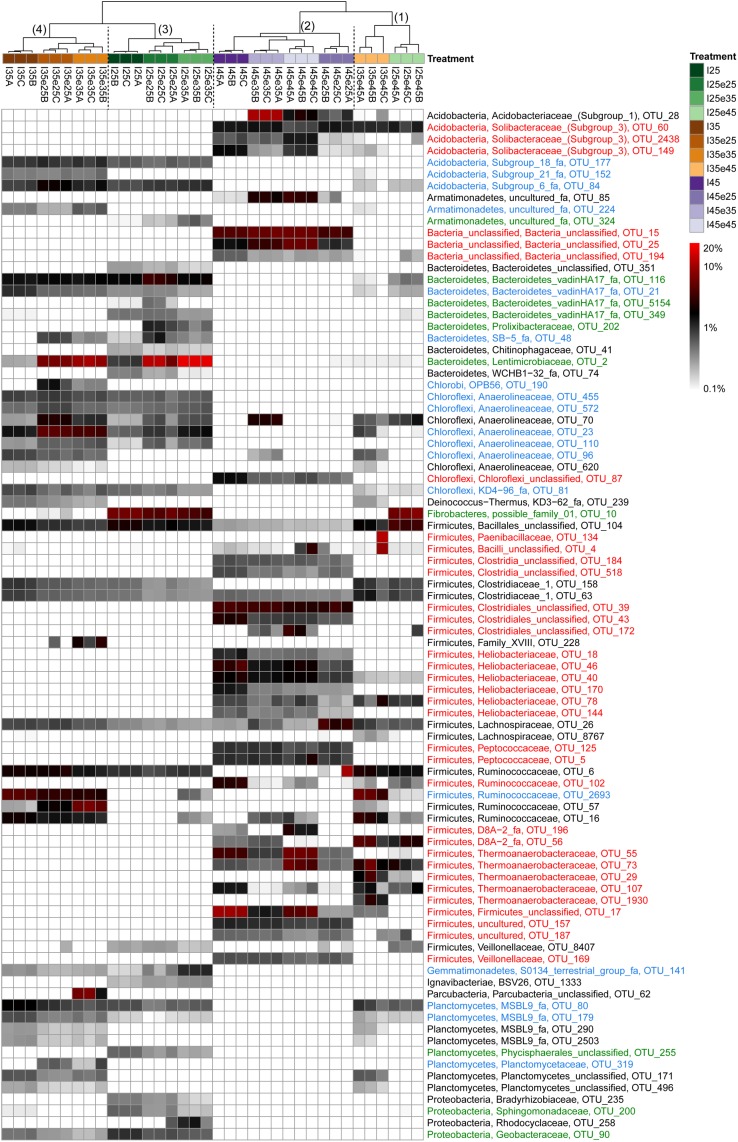
Relative abundances of selected bacterial OTUs that explain most of the differences between treatments in Italian paddy soil. Descriptions of OTUs selection, sample clustering and heatmap construction are given in Section “Materials and Methods.” See Section “Materials and Methods” in the main text and Supplementary Figure S1 for the description of each treatment. The color scale is logarithmic to emphasize rare taxa, with 0.1% being set as the lower bound for logarithmic transformation. Numbers above the dendrogram indicate clusters described in the main text. Names of the OTUs were colored according to the three major modules in the Supplementary Figure S8, i.e., green, blue, and red corresponding to module 25, 35, and 45°C, respectively (see Supplementary Figure S8). OTUs in black were not in the network.

**FIGURE 7:**
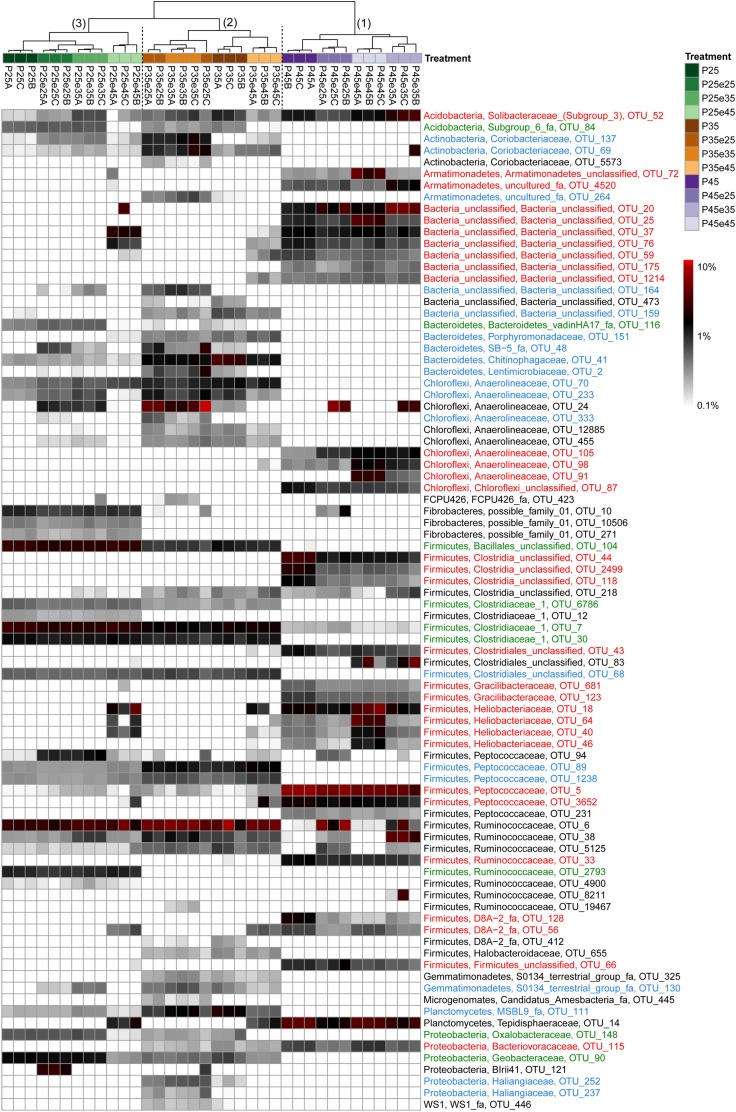
Relative abundances of selected bacterial OTUs that explain most of the differences between treatments in the Philippines paddy soils. Names of the OTUs were colored according to the three major modules in the Supplementary Figure S9, i.e., green, blue, and red corresponding to module 25, 35, and 45°C, respectively (see Supplementary Figure S9). Refer to the legend of Figure 6 for additional details.

**FIGURE 8:**
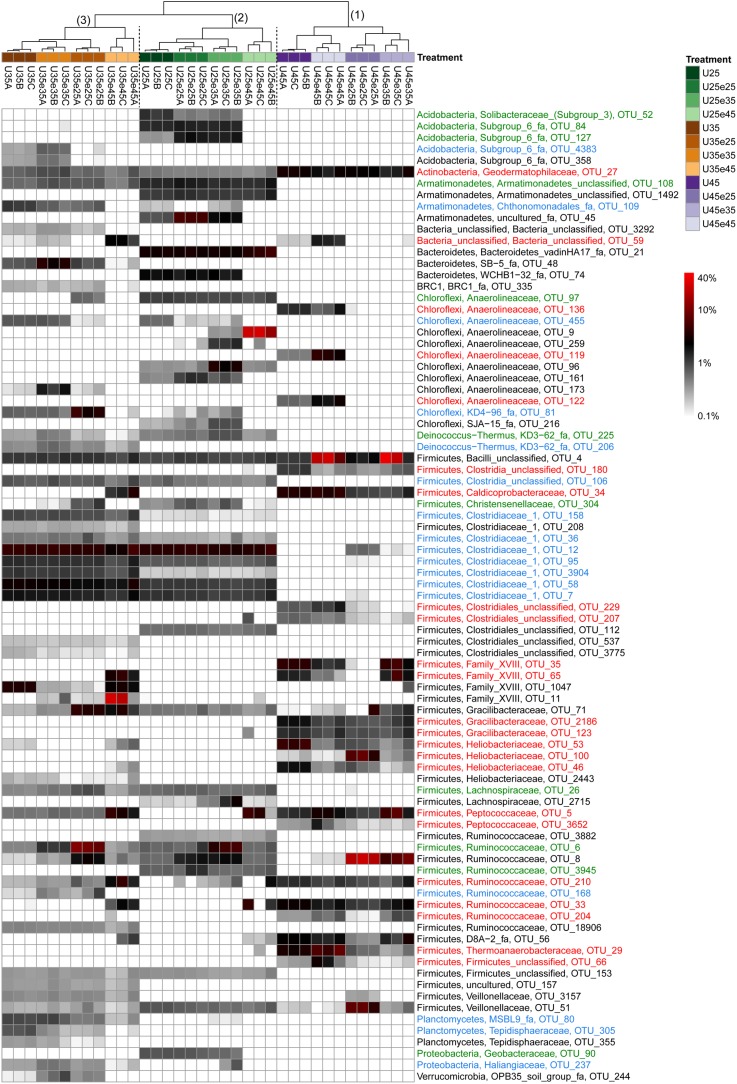
Relative abundances of selected bacterial OTUs that explain most of the differences between treatments in Utah desert soil. Names of the OTUs were colored according to the three major modules in the Supplementary Figure S10, i.e., green, blue, and red corresponding to module 25, 35, and 45°C, respectively (see Supplementary Figure S10). Refer to the legend of Figure 6 for additional details.

In order to identify the most important OTUs, 25 archaeal and 50 bacterial OTUs with the highest contributions in defining the first two PCA axes were selected and used to create a heatmap for each soil and for Archaea (Figure 5) and Bacteria (Figure 6–8), respectively. The heatmaps showed distinct clusters of the archaeal and bacterial community compositions. The clustering of samples in the heatmaps was similar to that of NMDS analysis (Supplementary Figures S6, S7) and differentiated the samples largely according to the temperatures used for incubation. In the Italian soil (Figure 5A, 6), four main clusters (from right to left) separated treatments with (1) pre-incubation at either 25 or 35°C followed by incubation at 45°C; (2) pre-incubation at 45°C followed by incubation at the same or other temperatures; (3) pre-incubation at 25°C followed by incubation at either 25 or 35°C; and (4) pre-incubation at 35°C followed by incubation at either 25 or 35°C. In the Philippines and Utah soils (Figure 5B,C, 7, 8), there were three main clusters, which showed a similar separation as in the Italian soil, with the first cluster containing the treatments with pre-incubation at 45°C, while the other two clusters contained the treatments with pre-incubations at either 25 or 35°C including the treatments that were later shifted to 45°C.

### Network Analysis of Microbial Operational Taxonomic Units

The combined archaeal and bacterial OTUs of each soil were used for network analysis. Each soil exhibited three major modules (Supplementary Figures S8–S10). Most of the OTUs enriched in the cluster with pre-incubation at 45°C of the archaeal and bacterial heatmaps (shown in red in Figure 5–8) were found in one of the three major modules (45°C) of the networks (Supplementary Figures S8–S10). The OTUs enriched in clusters with pre-incubation at 25 and 35°C (shown in green and blue, respectively, in Figure 5–8) also formed two network modules (see also Supplementary Datasets 1–3 for details), but each of these modules contained OTUs from both 25 and 35°C (Supplementary Figures S8–S10). Hence these two network modules did not clearly separate between the two moderate temperatures. Interestingly, there were only a few among these OTUs that were shared between all the different soils, i.e., *Peptococcaceae* OTU_5 and *Heliobacteriaceae* OTU_46 for the 45°C network module, and *Geobacteraceae* OTU_90 and *Acidobacteria* OTU_84 for the two moderate temperature network modules (Supplementary Figures S8–S10). There were also only few OTUs that were shared between two soils. The same was true for archaeal OTUs. Hence, most of the OTUs found in both network and heatmap were unique for each soil.

The assembly of OTUs from different functional groups into modules were largely different. For instance, OTUs of hydrogenotrophic methanogens (*Methanocellaceae* and/or *Methanobacteriaceae*) occurred in network modules of either 45 or 35°C but not 25°C (Figure 5 and Supplementary Figures S8–S10), while OTUs of acetoclastic methanogens (*Methanosarcinaceae* and/or *Methanobacteriaceae*) occurred only at modules of moderate temperatures (either 25 or 35°C) (Figure 5 and Supplementary Figures S8–S10). There were also several archaeal OTUs, which did not belong to canonical methanogens, but occurred in both heatmap and one of the network modules, i.e., *Woesearchaeota* (626 and 1382), *Bathyarchaeota* (138, 384, 398, and 1354), *Thermoplasmata* (277), and SCG (77, 135, and 430). They also preferentially occurred at moderate temperatures modules (Figure 6–8, Supplementary Figures S8–S10, and Supplementary Datasets 1–3).

In addition, some OTUs from the same functional group showed preferences for network modules and distinct temperature responses. For example, there were four major *Methanocellaceae* OTUs (13, 31, 287, and 574) in Italian paddy soil which occurred in both heatmap and one of the network modules (Figure 5A and Supplementary Figure S8). One OTU (31) was exclusively found in soil that had experienced incubation at 45°C, two OTUs (287 and 574) exclusively at 35°C, and one OTU (13) in all treatments (Figure 5A). The same were true for some other methanogens (e.g., OTUs of *Methanobacteriaceae*) and bacteria from the same potential functional groups. Of all these bacterial OTUs, only those of *Thermoanaerobacteraceae* are clear candidates for the function as syntrophic acetate oxidizers (Liu and Conrad, 2010). Five *Thermoanaerobacteraceae* OTUs (55, 73, 29, 107, and 1930) occurred in Italian paddy soil only after experiencing incubation at 45°C (Figure 6). Although all five OTUs occurred in the same module, their responses to temperature were substantially different. The Philippines paddy soil, by contrast, did not contain a major *Thermoanaerobacteraceae* OTU (Figure 7), while the Utah desert soil only contained *Thermoanaerobacteracea* OTU_29 (Figure 8). Finally, the assembly into module and the temperature responses of the same OTU maybe differed between different soils. For example, *Methanosarcinaceae* OTU_42 showed the highest abundances at the two moderate temperatures and recovered when the temperature was changed back from 45°C to moderate temperatures in Italian soil. The relative abundances of OTU_42 were relatively stable in the Philippines soil (Figure 5B) while dramatically decreased in the Utah soil when pre-incubated at 45°C (Figure 5C). In addition, OTU_42 did not occur in the Italian soil network (Figure 5A and Supplementary Figure S8).

## Discussion

### Temperature Effects on the Soil Microbial Communities

The incubation temperature had a decisive effect on the composition of the methanogenic microbial communities and their function in the different soils. The most important functional differences were the pathways of acetate conversion and methanogenesis in Italian and Utah soil, which were characterized by the operation of acetoclastic methanogenesis and of syntrophic acetate oxidation coupled to hydrogenotrophic methanogenesis at mesophilic (25 and 35°C) versus thermophilic (45°C) incubation conditions, respectively. In the Philippines soil, by contrast, acetoclastic methanogenesis operated at both mesophilic and thermophilic conditions. These temperature-dependent functional differences, which are mainly seen from the different fractionation of ^13^C, have been discussed in detail in our previous publication (Liu et al., 2018). Our present study confirms and extends these observations with the results of factorial temperature shifts. Taken together, both studies allowed network analysis of the combined archaeal and bacterial microbial communities demonstrating that incubation temperature not only affected the function but also the composition of the methanogenic microbial communities. This analysis showed a distinct network module for each soil when incubated at 45°C. There were two additional network modules for moderate temperatures, albeit not clearly separated with respect to 25°C versus 35°C. However, in NMDS and heatmap analyses each single temperature treatment had a different effect. Nevertheless, the most drastic differences were between soils treated at moderate (25 and 35°C) versus elevated (45°C) temperatures, as expected from previous experiments (Liu et al., 2018). However, even within these big clusters of community responses there was a distinct sub-clustering, sometimes distinguishing every single treatment. For example, shifts between incubations at 25°C versus 35°C often resulted in different compositions of the archaeal and the bacterial community. Interestingly, the temperature response of archaeal versus bacterial communities resulted in an almost identical pattern of clustering. By contrast, a temperature interval of only 5°C in wetland soil from the Tibetan Plateau showed no effect on pathway and community composition of methanogenic archaea (Cui et al., 2015). Our results show that temperature at sufficiently large intervals is a very important regulator of community composition (Conrad, 2008; Roussel et al., 2015). They also show that there was a substantial taxonomic richness within the different functional groups (e.g., hydrogenotrophic methanogenesis; see discussion below) indicating that temperature is an important environmental effector causing the emergence of functional redundancy (Louca et al., 2018).

The incubation temperature also affected the size of the microbial populations, but only to a relatively small extent. The archaeal and bacterial counts (given as 16S rRNA gene copy numbers) always were in a reasonable range of 10^8^ to 10^10^ per gram soil with Bacteria usually being by a factor of 10 more numerous than archaea. Higher bacterial than of archaeal counts have commonly been observed (Fernandez Scavino et al., 2013; Breidenbach et al., 2017; Reim et al., 2017). Numbers apparently increased from the pre-incubation to the subsequent incubation indicating growth of microbial populations. This observation is reasonable, since the soil had been amended with cellulose as additional substrate to equalize substrate availability across the different soils. As microbial growth occurred at the different temperatures, the individual bacterial and archaeal populations were apparently affected to different extents, thus resulting in different community compositions.

Temperature also affected the overall diversity of the microbial communities. In particular, diversity in Italian paddy soil and Utah desert soil was generally lower when the soil had been incubated at 45°C compared to soil incubated at moderate temperatures only. Such decrease of diversity was found for both Archaea and Bacteria, which was not consistently reversible, when the temperature was changed back from 45°C to moderate conditions. We assume that the time for recovery was not sufficient, since microbial counts were not consistently higher when temperature was shifted back from 45°C to moderate conditions. An earlier study with Italian paddy soil indicated that recovery might be possible when the soil is in addition inoculated with soil from moderate temperatures (Noll et al., 2010). Therefore, since we did not use inoculation in our experiments, we assume that recovery was not possible since the microbes had been killed by exposure to 45°C. In the Philippines paddy soil, decrease of diversity was observed for Bacteria, while archaeal diversity was not so much affected.

### Temperature Modulation of Important Microbial OTUs

Our study demonstrates the modulation of the microbial community composition by temperature in three different soils. This intrinsic ability of the soil methanogenic microbial communities is remarkable. Although elevated temperatures, also well beyond 45°C, do occur in tropical soils, they are probably not reached during the time when the soils are flooded and the methanogenic communities are active. The fact, that the soils nevertheless possess populations of moderately thermophilic Bacteria and Archaea indicates that the non-flooded conditions might also affect particular microbial populations.

It is difficult to deduce the importance of microbial species for specific functions, since these are not or not exactly known for most of the detected OTUs. For methanogenic microbial communities, however, some connection can be made, since we are relatively sure about the physiological capacities of many methanogenic archaeal taxa. Our study revealed among acetoclastic (especially *Methanotrichaceae*) and hydrogenotrophic (especially *Methanocellaceae*) methanogenic archaea quite some redundancy with respect to different taxa (OTUs), some of them responding differently upon temperature change, others perhaps contributing to functional redundancy (Louca et al., 2018).

Acetoclastic methanogenesis is usually the process that is responsible for acetate consumption during the methanogenic degradation of organic matter. In the different soils, acetoclastic methanogenesis (as identified by relatively low α_app_ values) only operated when putative acetoclastic archaeal taxa (*Methanosarcinaceae*, *Methanotrichaceae*) were present. The *Methanotrichaceae* in Italian paddy soil were strictly mesophilic and could only serve the acetoclastic function at moderate temperatures. This result is consistent with previous experiments, albeit they did not extend to the OTU level (Conrad et al., 2009). The *Methanotrichaceae* were represented by 8 different OTUs with relative abundance larger than 0.8%, thus presenting a substantial functional redundancy. Many of the OTUs were organized in one of the network modules indicating that their functioning as acetoclastic methanogens may be affected by interactions with other microbes. The acetoclastic archaeal community in Italian paddy soil consisted in addition of one major *Methanosarcinaceae* OTU_42. Similarly to the *Methanotrichaceae* this OTU was missing whenever the soil was incubated at 45°C, but recovered when the incubation temperature was again decreased to moderate conditions. The recovery of *Methanosarcinaceae* OTU_42 after temperature shift from 45 to 25°C or 35°C indeed resulted in a gradual recovery of the acetoclastic function as seen by the decrease of α_app_ values (Figure 2C). The shape of the α_app_ curves possibly indicates the temporal recovery of the acetoclastic *Methanosarcina*, which happened earlier at 35°C than at 25°C (Figure 2C).

The acetoclastic archaeal community in the Philippines paddy soil consisted of one major *Methanotrichaceae* OTU_205 and one *Methanosarcinaceae* OTU_42, which both occurred also in the Italian paddy soil. Hence there was little functional redundancy in this soil. It is noteworthy that these two acetoclastic methanogens were thermotolerant in the Philippines soil, but were not thermotolerant in the Italian soil. We conclude that *Methanotrichaceae* OTU_205 and *Methanosarcinaceae* OTU_42 exist as taxonomically closely related thermotolerant and thermointolerant ecotype. The existence of such ecotypes is commonly found in the literature (Simankova et al., 2003; Acinas et al., 2004; Wu et al., 2006; Becraft et al., 2011). Interestingly, the Utah desert soil also contained *Methanosarcinaceae* OTU_42, but like the Italian soil only as thermointolerant ecotype. This OTU was the sole acetoclastic methanogen in Utah soil, which thus showed no functional redundancy. Although it occurred at moderate temperatures, its population strongly decreased after pre-incubation at 45°C (Figure 5C), so that acetate was no longer consumed and accumulated (Supplementary Table S2).

The major hydrogenotrophic taxa in Italian paddy soil were *Methanobacteriaceae* and *Methanocellaceae*. *Methanosarcinaceae* can also use H_2_/CO_2_ as substrate, albeit not as efficiently as *Methanobacteriaceae* and *Methanocellaceae* (Thauer et al., 2008). Among the *Methanocellaceae* there was one OTU (31), which was exclusively found at 45°C, while another OTU (13) was found at all temperatures. In the Philippines paddy soil, however, this OTU (13) only occurred, when the soil had not been pre-incubated at 45°C, again indicating the existence of ecotypes. In Utah desert soil, there were two *Methanocellaceae* OTUs (287, 3964), but not at elevated temperatures. Only one *Methanocellales* OTU_50 also occurred at 45°C. However, the more important hydrogenotrophic methanogenic taxon at 45°C was *Methanobacteriaceae* OTU_3, which was also found in Italian paddy soil. A second *Methanobacteriaceae* OTU_1034, which was also found in Italian and the Philippines paddy soils, was less abundant and seemed to prefer moderate temperatures. Hence, hydrogenotrophic methanogens were functionally redundant.

*Bathyarchaeota* were found in all three soils with 2–3 different OTUs. *Bathyarchaeota*, which belong to the TACK superphylum (Adam et al., 2017; Spang et al., 2017) have not yet been cultured. Although metagenomic data suggest that they may be potential methanogens (Evans et al., 2015; Spang et al., 2017), other physiological functions are also conceivable (He et al., 2016; Spang et al., 2017; Xiang et al., 2017). One OTU (138) occurred in all three soils, but only at moderate temperatures. Potentially thermophilic OTUs only occurred in the Philippines paddy soil, with unknown function. *Woesearchaeota*, which belong to the DPANN superphylum, were found in the Italian and Philippines paddy soils at moderate temperatures. However, their functions are unknown, albeit probably not methanogenic (Adam et al., 2017; Spang et al., 2017). The SCG archaea, which occurred as several OTUs in all the soils at all temperatures most probably did also not function as methanogens (Lu et al., 2017).

If acetoclastic methanogenesis does not operate in methanogenic systems, acetate consumption can only be achieved by acetate-oxidizing bacteria that are syntrophically coupled to hydrogenotrophic methanogens. We identified the operation of syntrophic acetate oxidation by rather high α_app_ values and the fact that acetate did not accumulate. Such conditions happened in Italian paddy soil, when the temperature was increased to 45°C as reported before (Conrad et al., 2009). Syntrophic *Thermoanaerobacteraceae* have been identified as being responsible for acetate consumption under these conditions (Liu and Conrad, 2010). *Thermoanaerobacteraceae* were also found in our experiments. The relative abundance of this taxon (represented by 4 major OTUs) specifically increased whenever the soil was incubated at 45°C. The Philippines paddy soil, which displayed acetoclastic methanogenesis at all the different temperatures, did not contain a major *Thermoanaerobacteraceae* OTU. However, the Utah desert soil contained *Thermoanaerobacteracea* OTU_29, which was identical to the OTU identified in Italian paddy soil. The failed instantaneous replacement of acetoclastic methanogenesis in Utah soil by syntrophic acetate oxidizers resulted in Utah soil in decrease of methanogenic activity and accumulation of acetate.

Besides the putatively acetate-oxidizing *Thermoanaero-bacteraceae* temperature effects were also found for other Bacteria. The functions of these different taxa within the methanogenic degradation process are unclear, but most probably involve various hydrolytic and fermentation processes. *Helicobacteraceae*, for example, may be involved in anaerobic oxidation of propionate, possibly even acetate (Noll et al., 2010; Sattley and Madigan, 2014; Liu et al., 2018; Peng et al., 2018). The different bacterial taxa were separated together with archaeal taxa into three major network modules, the most distinct being those in soils exposed to 45°C versus those exposed to moderate temperatures (either 25 or 35°C). Whereas elevated temperature (45°C) seemed to favor *Heliobacteriaceae* OTUs in all three soils, and *Peptococcaceae* OTUs in particular in the Philippines soil, moderate temperatures seemed to generally favor *Anaerolineaceae* and *Ruminococcaceae* OTUs. There were only four bacterial OTUs that were shared between all of the soils, two occurring in the network modules at 45°C and two in those at 25 or 35°C. These OTUs belonged to *Heliobacteriaceae*, *Peptococcaceae*, *Geobacteraceae*, and *Acidobacteria*, respectively. Besides this core of bacterial taxa, each soil possessed its own characteristic bacterial community. There were numerous bacterial taxa that were affected by temperature specifically in each soil. In general, soils apparently had their individual microbial communities, which reacted to temperature exposure.

## Conclusion

Our results demonstrated quite some taxonomic redundancy of the functional microbial groups, in particular syntrophic acetate oxidation and acetoclastic and hydrogenotrophic methanogenesis, after exposure to mesophilic (25 and 35°C) and thermophilic (45°C) temperatures. Our results also show that the methanogenic bacterial and archaeal communities were indeed organized in co-occurrence networks in the three different soils studied. This observation indicates that methanogenic soil microbial communities are organized in distinct temperature-dependent modules rather than in a random way.

Our results are consistent with our initial hypothesis that function is determined by the bacterial and/or archaeal community structures. The pathways of CH_4_ production changed between acetoclastic methanogenesis and syntrophic acetate oxidation, always combined with hydrogenotrophic methanogenesis. These changes were accompanied by shifts in community composition. These shifts were in particular seen by changes in the relative abundance of particular OTUs belonging to hydrogenotrophic and acetoclastic methanogenic taxa and to potentially syntrophic acetate-oxidizing *Thermoanaerobacteraceae*. However, there were also changes in other bacterial taxa that were probably involved in hydrolytic and fermentation processes.

Our results also supported our second hypothesis that temperature affects the taxonomic structure within the different functional groups. There was a substantial taxonomic redundancy allowing for the different functions within the methanogenic pathways (Louca et al., 2018). We also realized an amazing individuality of the different soils with respect to microbial OTUs, which were hardly shared. Even within shared OTUs we realized different adaptabilities to temperature change suggesting the existence of ecotypes within the same OTU.

Thirdly, we realized that the overall microbial diversity was reduced at 45°C. Once the diversity was lost, it was not fully regenerated by reversal of incubation temperatures, probably since the microbial seedbank was no longer available due to laboratory confinement. In summary, our experiments showed that temperature was an important regulator for structure and function of methanogenic microbial communities, which in all the soils tested displayed distinct temperature modules within co-occurrence networks.

Nevertheless, taxonomic microbial community structures in each soil were different and reacted individually to temperature treatment for reasons that are presently not understood. In particular it is unclear, which factors allow soils possessing or not possessing thermophilic populations of acetoclastic methanogens. The mechanistic basis for why structure and function of the methanogenic microbial communities drastically changed at around 45°C, and not at 25 or 35°C, is also unknown. We think that future research should consider the observation that acetate consumption by the archaeal acetoclastic methanogenic pathway is replaced by the bacterial Wood–Ljungdahl pathway. It should also consider the fact that Bacteria and Archaea possess different membrane lipids with inherently different functional constraints (Valentine, 2007).

## Data Availability

The datasets generated for this study can be found in NCBI Sequence Read Archive, SRP133538.

## Author Contributions

PL performed the data analysis. MK carried out the experiments. PL and RC wrote the manuscript.

## Conflict of Interest Statement

The authors declare that the research was conducted in the absence of any commercial or financial relationships that could be construed as a potential conflict of interest.

## Footnotes

1 https://drive5.com/python/

2 http://www.r-project.org/

3 https://gephi.github.io/
