# Supplementary material for: Temperature-Dependent Network Modules of Soil Methanogenic Bacterial and Archaeal Communities
Source: Front Microbiol. 2019 Mar 12;10:496. doi: 10.3389/fmicb.2019.00496 (PMC6422946; doi:10.3389/fmicb.2019.00496)
Supplement: Supplementary file 1 [file Data_Sheet_1.PDF]

Temperature-dependent network modules of soil methanogenic bacterial and archaeal communities

Pengfei Liu, Melanie Klose, Ralf Conrad

Supplementary Information

This file contains:

1, Supplementary Tables

2, Supplementary Figures

1, Supplementary Tables

Table S1. Barcodes for sequencing in the current study

Barcode Number	Barcode sequence (5'-3')	End of pre-incubation	End of incubation		
		Library1	Library2 (Italy)	Library3 (the Philippines)	Library4 (Utah)
4000	ACACGT	U25A*	I25e25A	P25e25A	U25e25A
4001	ACGTAC	U25B*	I25e25B	P25e25B	U25e25B
4002	ACTGCA	U25C*	I25e25C	P25e25C	U25e25C
4003	AGAGTC	U35A	I35e25A	P35e25A	U35e25A
4004	AGCTGA	U35B	I35e25B	P35e25B	U35e25B
4005	AGTCAG	U35C	I35e25C	P35e25C	U35e25C
4006	ATATCG	U45A	I45e25A	P45e25A	U45e25A
4007	ATCGAT	U45B	I45e25B	P45e25B	U45e25B
4008	ATGCTA	U45C	I45e25C	P45e25C	U45e25C
4009	CACAGT	I25A	I25e35A	P25e35A	U25e35A
4010	CAGTCA	I25B	I25e35B	P25e35B	U25e35B
4011	CATGAC	I25C	I25e35C	P25e35C	U25e35C
4012	CGATAT	I35A	I35e35A	P35e35A	U35e35A
4013	CGCGCG	I35B	I35e35B	P35e35B	U35e35B
4014	CGTATA	I35C	I35e35C	P35e35C	U35e35C
4015	GACTAG	I45A	I45e35A	P45e35A	U45e35A
4016	GAGATC	I45B	I45e35B	P45e35B	U45e35B
4017	GATCGA	I45C	I45e35C	P45e35C	U45e35C
4018	GTACAC	P25A	I25e45A	P25e45A	U25e45A
4019	GTCACA	P25B	I25e45B	P25e45B	U25e45B
4020	GTGTGT	P25C	I25e45C	P25e45C	U25e45C
4021	TACGTA	P35A	I35e45A	P35e45A	U35e45A
4022	TAGCAT	P35B	I35e45B	P35e45B	U35e45B
4023	TATACG	P35C	I35e45C	P35e45C	U35e45C
4024	TCAGAG	P45A	I45e45A	P45e45A	U45e45A
4025	TCGAGA	P45B	I45e45B	P45e45B	U45e45B
4026	TCTCTC	P45C	I45e45C	P45e45C	U45e45C

Note:

PCR primers (515F, 5’-GTGCCAGCMGCCGCGGTAA-3’ and 806R, 5’- GGACTACVSGGGTATCTAAT -3’) targeting the V4 region of the 16S rRNA gene (approximately 250 nucleotides) for both archaea and bacteria were used (Bates et al., 2011). For sequencing, a 16 bp sequencing adaptor and 6 bp barcode were integrated ahead of the forward primer [5’-GATGGCCATTACGGCC-(6 bp barcode)-515F-3’] and a 16 bp sequencing adaptor was integrated ahead of the reverse primer [5’-GATGGCCATTACGGCC-806R-3’]. In total, twenty-seven forward primers containing specific barcode sequences were synthesized.

*, A, B and C indicate replicates. I, P, and U stand for Italian, the Philippines and Utah soil, respectively.

Table S2. The concentrations (mM) of volatile fatty acids at the end of incubation in different treatments of Italian, the Philippines and Utah soil, respectively.

Phase	Treatment	Lactate	Formate	Acetate	Propionate	Butyrate	Iso-valerate	Valerate	Capronate
Pre-incubation	I25	-	0.205 (0.069)	0.021 (0.003)	-	-	-	-	-
	I35	-	0.409*	0.017 (0.005)	-	-	-	-	-
	I45	-	0.057 (0.023)	0.122 (0.053)	-	-	-	-	-
Incubation	I25e25	-			-	-	-	-	-
	I25e35	-			-	-	-	-	-
	I25e45	-	-	0.833 (0.515)	-	-	-	-	0.165 (0.088)
	I35e25	-			-	-	-	-	-
	I35e35	-			-	-	-	-	-
	I35e45	-	0.025*	0.85 (0.44)	0.133 (0.039)	-	-	-	-
	I45e25	-	0.023 (0.004)	0.037 (0.019)	0.261 (0.222)	-	-	-	-
	I45e35	-	-	-	-	-	-	-	-
	I45e45	-	0.027*	0.028 (0.006)	-	-	-	-	-
Pre-incubation	P25	-	-	-	-	-	-	-	-
	P35	-	-	-	-	-	-	-	-
	P45	-	0.067*	0.049 (0.031)	-	-	-	-	-
Incubation	P25e25	-	-	-	-	-	-	-	-
	P25e35	-	-	-	-	-	-	-	-
	P25e45	-	-	0.04 (0.01)	-	-	-	-	-
	P35e25	-	-	-	-	-	-	-	-
	P35e35	-	-	-	-	-	-	-	-
	P35e45	-	-	0.048 (0.035)	-	-	-	-	-
	P45e25	-	-	-	-	-	-	-	-
	P45e35	-	-	-	-	-	-	-	-
	P45e45	-	-	-	-	-	-	-	-
Pre-incubation	U25	0.012*	0.189 (0.074)	0.052 (0.022)	0.012*	-	-	-	0.269*
	U35	0.011 (0.003)	0.103 (0.073)	0.073 (0.064)	0.014*	-	-	-	-
	**U45**	0.131 (0.057)	0.353 (0.145)	**4.144 (1.459)**	0.365 (0.364)	0.388 (0.142)	1.762 (0.317)	0.053 (0.029)	0.92 (0.108)
Incubation	U25e25	0.007*	0.039 (0.014)	0.055 (0.037)	-	-	-	-	-
	U25e35	0.004*	0.021 (0.002)	0.033 (0.003)	-	0.126*	-	-	0.034*
	**U25e45**	-	-	**1.843** (0.55)	0.199 (0.225)	0.037*	0.363 (0.149)	0.016*	0.326 (0.104)
	U35e25	-	-	0.053 (0.026)	1.91 (1.422)	-	0.078 (0.043)	-	0.055*
	U35e35	0.006*	0.04*	0.03 (0.011)	-	-	-	-	0.082 (0.021)
	**U35e45**	-	0.11*	**1.878** (1.261)	0.337 (0.381)	0.076 (0.015)	0.198 (0.15)	-	0.152 (0.029)
	**U45e25**	0.091 (0.025)	0.083 (0.033)	**75.512 (3.442)**	2.355 (0.299)	3.954 (1.339)	1.571 (0.098)	0.061 (0.013)	0.775 (0.07)
	**U45e35**	0.085 (0.006)	0.057 (0.002)	**54.801 (8.585)**	1.576 (0.115)	3.06 (0.637)	1.638 (0.061)	0.063 (0.008)	0.707 (0.03)
	**U45e45**	0.087 (0.021)	0.093 (0.063)	**6.535 (1.048)**	0.527 (0.247)	0.154 (0.019)	1.425 (0.169)	0.032 (0.022)	0.932 (0.066)

Note:

Data shown are mean (standard deviations), with n=3.

Data from the pre-incubation (I25, 35, 45, P25, 35, 45 and U25, 35, 45) were reported in our previous paper (Liu et al., 2018, Soil Biology and Biochemistry, 124,236-244).

*, detected in only one of the replicates. -, not detected in all triplicates.

Table S3. Sample summary, operational taxonomic unit (OTU) and alpha diversity indices of archaeal 16S rRNA gene sequences. Data are means of triplicates per treatment, with standard deviations in parentheses.

Treat-ments	Total reads No.[1]	No. of OTUs [1]	Coverage[2]	alpha diversity index[2] Richness[3]	Shannon[4]	Fisher[5]	Evenness[6]
Italy							
I25	24074 (1838)	215 (7)	0.9912 (0.0011)	394 (50) a	3.32 (0.01) a	51.3 (0.5) a	0.592 (0.002) ab
I35	29142 (3512)	207 (3)	0.9911 (0.0013)	396 (58) a	3.34 (0.16) a	44.7 (2.8) bc	0.607 (0.026) a
I45	73527 (16447)	89 (8)	0.9961 (0.0005)	151 (26) d	1.61 (0.09) f	13.3 (0.6) g	0.358 (0.021) e
I25e25	12182 (1844)	175 (6)	0.9925 (0.0004)	327 (14) b	3.2 (0.04) ab	45.7 (2.7) b	0.58 (0.005) bc
I25e35	23533 (4521)	178 (6)	0.9925 (0.0008)	321 (6) b	3.13 (0.05) bc	41.1 (2.4) c	0.541 (0.009) c
I25e45	55914 (25493)	128 (7)	0.9948 (0.001)	204 (30) c	1.92 (0.34) e	24.3 (3.4) e	0.367 (0.022) e
I35e25	30997 (7610)	155 (4)	0.9922 (0.0008)	343 (29) ab	2.86 (0.03) cd	34.9 (1.9) d	0.576 (0.013) cd
I35e35	40112 (2814)	157 (2)	0.993 (0.0011)	291 (41) b	2.82 (0.06) d	34.8 (4.1) d	0.533 (0.01) d
I35e45	77416 (19713)	128 (13)	0.9944 (0.0001)	215 (8) c	1.69 (0.26) ef	21.2 (1.9) e	0.378 (0.002) e
I45e25	53200 (25072)	67 (12)	0.9966 (0.0004)	138 (25) d	1.63 (0.11) ef	12.9 (0.4) g	0.384 (0.06) e
I45e35	69618 (20491)	64 (11)	0.9965 (0.0004)	141 (17) d	1.71 (0.01) ef	14 (0.4) f	0.346 (0.048) e
I45e45	80450 (34201)	65 (5)	0.9977 (0.0006)	102 (38) d	1.02 (0.12) g	7.8 (1.2) h	0.252 (0.02) f
the Philippines							
P25	35489 (3477)	153 (3)	0.9938 (0.001)	270 (42) abc	2.81 (0.02) def	31.7 (1.6) bc	0.538 (0.008) fg
P35	49911 (4883)	157 (7)	0.9938 (0.0004)	275 (23) a	3.09 (0.1) a	32.7 (1.3) ab	0.589 (0.017) ab
P45	45611 (5645)	102 (7)	0.9959 (0.0006)	179 (16) de	2.73 (0.06) fg	19.7 (2.8) ef	0.568 (0.008) de
P25e25	30281 (1151)	143 (10)	0.9942 (0.0006)	265 (25) ab	2.83 (0.02) de	32.6 (1.4) ab	0.539 (0.004) fg
P25e35	43584 (3003)	160 (4)	0.9933 (0.0008)	302 (42) a	2.93 (0.04) bc	35 (1.1) a	0.573 (0.018) ef
P25e45	33694 (5170)	107 (14)	0.9951 (0.0001)	224 (5) d	2.65 (0.04) gh	25 (1.5) de	0.485 (0.01) g
P35e25	43265 (5132)	145 (8)	0.9943 (0.0002)	267 (10) ab	2.98 (0.06) ab	31.5 (2.6) bc	0.552 (0.007) bcd
P35e35	48852 (885)	135 (14)	0.9958 (0.0019)	221 (61) bcd	3.03 (0.06) ab	28.6 (4.6) cd	0.592 (0.008) a
P35e45	45725 (4975)	111 (6)	0.9953 (0.0001)	231 (7) cd	2.86 (0.07) cd	24.4 (0.2) de	0.569 (0.016) cde
P45e25	49873 (10189)	76 (4)	0.9968 (0.0006)	151 (26) e	2.22 (0.02) h	14.7 (1.2) g	0.529 (0.003) g
P45e35	35234 (10242)	79 (7)	0.9968 (0.0002)	145 (11) e	2.65 (0.06) gh	16.4 (0.6) fg	0.573 (0.013) de
P45e45	38535 (5036)	84 (8)	0.9969 (0.0008)	150 (31) e	2.75 (0.07) ef	16.5 (1.1) fg	0.589 (0.01) abc
Utah							
U25	24619 (1252)	92 (2)	0.9951 (0.0003)	192 (5) ab	2.51 (0.06) b	21.5 (1.7) ab	0.513 (0.006) bc
U35	37398 (8418)	115 (3)	0.9947 (0.0009)	230 (33) a	2.74 (0.05) a	24.6 (0.5) a	0.548 (0.009) a
U45	41060 (2385)	77 (9)	0.9963 (0.0004)	181 (44) ab	1.57 (0.07) fg	11.7 (0.4) fg	0.359 (0.017) ef
U25e25	19189 (4620)	54 (5)	0.9961 (0.0004)	164 (30) b	2.32 (0.11) c	16.8 (1) e	0.496 (0.021) c
U25e35	36033 (8293)	62 (1)	0.9959 (0.0004)	158 (10) bc	2.1 (0.11) ef	17.3 (1.3) de	0.463 (0.013) de
U25e45	20393 (1933)	58 (2)	0.996 (0.0004)	179 (34) ab	2.34 (0.06) cd	16.8 (0.4) ef	0.208 (0.012) c
U35e25	35114 (9373)	72 (6)	0.9961 (0.0012)	163 (41) bc	2.21 (0.11) de	18.6 (2.3) cde	0.445 (0.017) d
U35e35	44165 (1871)	76 (3)	0.9955 (0.0003)	195 (33) ab	2.24 (0.17) cde	20.4 (2.4) bc	0.462 (0.025) d
U35e45	21361 (7636)	69 (3)	0.9963 (0.0002)	168 (15) b	2.54 (0.02) ab	18.7 (1) bcd	0.225 (0.026) ab
U45e25	33903 (2156)	42 (2)	0.9984 (0.0001)	71 (6) d	0.83 (0.05) i	7.5 (0.2) h	0.5 (0.011) h
U45e35	25263 (9120)	42 (2)	0.9979 (0.0003)	90 (14) cd	0.91 (0.11) hi	7.9 (0.4) gh	0.532 (0.005) gh
U45e45	27976 (13503)	43 (7)	0.9982 (0.0004)	78 (14) d	1.02 (0.05) gh	8.2 (0.7) gh	0.252 (0.016) fg

Note:

1, OTUs were defined at a sequence identity level of 97%. Data were calculated from sequences without singletons.

2, Coverage and alpha diversity indices were calculated from data including singletons by using vegan and phyloseq package in R. Different letters behind data indicate significant difference ($p < 0.05$) between different treatments within each soil. Coverage: Good's coverage estimator (Good, 1953).

3, Richness: Chao1 estimator (Chao, 1984).

4, Shannon: Shannon–Weaver index (Shannon and Weaver, 1998).

5, Fisher: Fisher's diversity index (Fisher et al., 1943).

6, Evenness: Pielou's evenness index J (Pielou, 1975).

Table S4. Sample summary, operational taxonomic unit (OTU) and alpha diversity indices of bacterial 16S rRNA gene sequences. Data are means of triplicates per treatment, with standard deviations in parentheses.

				alpha diversity index [2]			
Treatments	Total reads No.[1]	OTU No.[1]	Good's coverage [2]	Richness [3]	Shannon [4]	Fisher [5]	Evenness [6]
Italy							
I25	468147 (6000)	9235 (35)	0.986 (0.0002)	16651 (319) a	7.07 (0.01) a	2384 (18) a	0.757 (0) a
I35	463021 (25125)	7680 (229)	0.9888 (0.0009)	13035 (893) b	6.71 (0.01) ab	1797 (81) c	0.737 (0.003) ab
I45	416167 (9743)	5493 (79)	0.9907 (0.0002)	10061 (247) e	5.36 (0.03) fg	1176 (28) g	0.61 (0.002) ef
I25e25	363785 (34169)	7596 (78)	0.9881 (0)	14081 (248) a	6.44 (0.25) ab	1951 (63) b	0.702 (0.026) bc
I25e35	453335 (28215)	7410 (88)	0.9887 (0.0001)	13143 (210) b	5.97 (0.04) de	1760 (12) c	0.682 (0.001) d
I25e45	341268 (28959)	6496 (177)	0.9898 (0.0001)	11843 (78) c	6.27 (0.04) bc	1597 (31) d	0.616 (0.015) bc
I35e25	376659 (29163)	6184 (94)	0.9898 (0.0001)	11734 (169) c	6.11 (0.01) cd	1475 (4) e	0.656 (0.005) cd
I35e35	433126 (56291)	6088 (219)	0.9908 (0.0004)	10642 (344) d	5.77 (0.17) ef	1349 (28) f	0.65 (0.02) d
I35e45	333188 (15810)	5357 (452)	0.9913 (0.0005)	9872 (743) e	5.76 (0.41) ef	1256 (142) g	0.612 (0.006) d
I45e25	344046 (34452)	4293 (223)	0.9925 (0.0003)	8323 (270) fg	5.29 (0.13) g	945 (38) i	0.695 (0.004) e
I45e35	326856 (20100)	4401 (103)	0.9921 (0.0001)	8683 (143) f	5.29 (0.06) gh	998 (18) h	0.653 (0.04) e
I45e45	306061 (21074)	4089 (107)	0.9925 (0.0002)	8198 (232) g	5.09 (0.11) h	927 (13) i	0.593 (0.012) f
The Philippines							
P25	470567 (51309)	8542 (294)	0.9895 (0.0005)	13079 (450) a	6.88 (0.02) a	1973 (49) a	0.749 (0.003) a
P35	486908 (30153)	7350 (236)	0.9905 (0.0006)	11428 (512) cd	6.49 (0.27) bc	1588 (93) cd	0.719 (0.028) bc
P45	486786 (14910)	5718 (120)	0.9929 (0.0004)	8534 (221) ef	5.74 (0.04) e	1122 (22) f	0.657 (0.006) de
P25e25	447443 (52566)	7826 (146)	0.9903 (0.0003)	12097 (269) ab	6.78 (0.03) ab	1790 (20) ab	0.744 (0.004) a
P25e35	505683 (69838)	7785 (215)	0.9905 (0.0002)	11813 (267) bc	6.74 (0.02) b	1731 (8) bc	0.692 (0.025) ab
P25e45	460133 (16726)	5790 (445)	0.9939 (0.0015)	8239 (1147) fg	6.18 (0.05) d	1195 (115) f	0.624 (0.006) c
P35e25	424553 (13790)	6739 (262)	0.9912 (0.0009)	10600 (608) d	6.2 (0.21) d	1480 (76) de	0.741 (0.002) cd
P35e35	468603 (26457)	6294 (336)	0.9928 (0.0013)	9277 (1034) e	6.31 (0.09) cd	1314 (110) e	0.712 (0.014) c
P35e45	464210 (14384)	5380 (334)	0.9947 (0.0009)	7466 (725) gh	6.17 (0.07) d	1087 (94) f	0.618 (0.013) c
P45e25	412419 (30713)	4569 (209)	0.9941 (0.0003)	6950 (327) hi	5.33 (0.05) ef	890 (37) g	0.704 (0.011) ef
P45e35	425697 (5308)	4414 (283)	0.9944 (0.0003)	6675 (431) ij	5.24 (0.16) f	835 (85) gh	0.709 (0.013) f
P45e45	480105 (25640)	4316 (61)	0.9951 (0.0004)	6143 (302) j	5.33 (0.07) ef	797 (14) h	0.63 (0.01) ef
Utah							
U25	474825 (59252)	6336 (270)	0.9894 (0.0002)	11699 (198) a	5.87 (0.01) a	1397 (16) a	0.658 (0) a
U35	485011 (36596)	5293 (126)	0.9909 (0.0001)	9836 (155) ab	5.46 (0.03) ab	1076 (16) ab	0.628 (0.003) cd
U45	444840 (35599)	4444 (258)	0.9921 (0.0004)	8307 (400) bc	4.66 (0.05) de	857 (41) de	0.548 (0.003) ef
U25e25	352843 (11362)	4069 (116)	0.993 (0.0003)	8008 (384) c	5.4 (0.03) bc	931 (25) bc	0.629 (0.002) bcd
U25e35	380434 (15816)	4015 (29)	0.9932 (0.0002)	7804 (322) c	5.49 (0.03) a	895 (14) cd	0.603 (0.009) ab
U25e45	388903 (14981)	3230 (186)	0.9945 (0.0003)	6169 (401) de	4.32 (0.38) efg	665 (55) g	0.487 (0.018) fg
U35e25	346356 (16430)	3262 (74)	0.9943 (0.0002)	6499 (122) d	5.04 (0.09) cd	719 (24) fg	0.642 (0.003) de
U35e35	397114 (23716)	3618 (62)	0.994 (0.0005)	6818 (514) d	5.38 (0.05) bc	774 (43) ef	0.638 (0.007) abc
U35e45	415317 (6246)	2929 (337)	0.9949 (0.0004)	5787 (396) ef	4.38 (0.51) ef	590 (84) h	0.473 (0.073) fg
U45e25	330485 (46891)	2626 (89)	0.9952 (0.0002)	5209 (207) fg	3.97 (0.14) fg	551 (11) h	0.52 (0.042) g
U45e35	358554 (57600)	2503 (184)	0.9954 (0.0005)	4999 (604) g	3.83 (0.65) g	513 (85) h	0.534 (0.055) g
U45e45	392180 (20831)	2603 (217)	0.9953 (0.0004)	5086 (494) g	3.97 (0.37) fg	513 (56) h	0.491 (0.04) g

Note:

1, OTUs were defined at a sequence identity level of 97%. Data were calculated from sequences without singletons.

2, Coverage and alpha diversity indices were calculated from data including singletons by using vegan and phyloseq package in R. Different letters behind data indicate significant difference ($p < 0.05$) between different treatments within each soil. Coverage: Good’s coverage estimator (Good, 1953).

3, Richness: Chao1 estimator (Chao, 1984).

4, Shannon: Shannon–Weaver index (Shannon and Weaver, 1949).

5, Fisher: Fisher's diversity index (Fisher, 1943).

6, Evenness: Pielou's evenness index J (Pielou, 1975).

Supplementary Figures

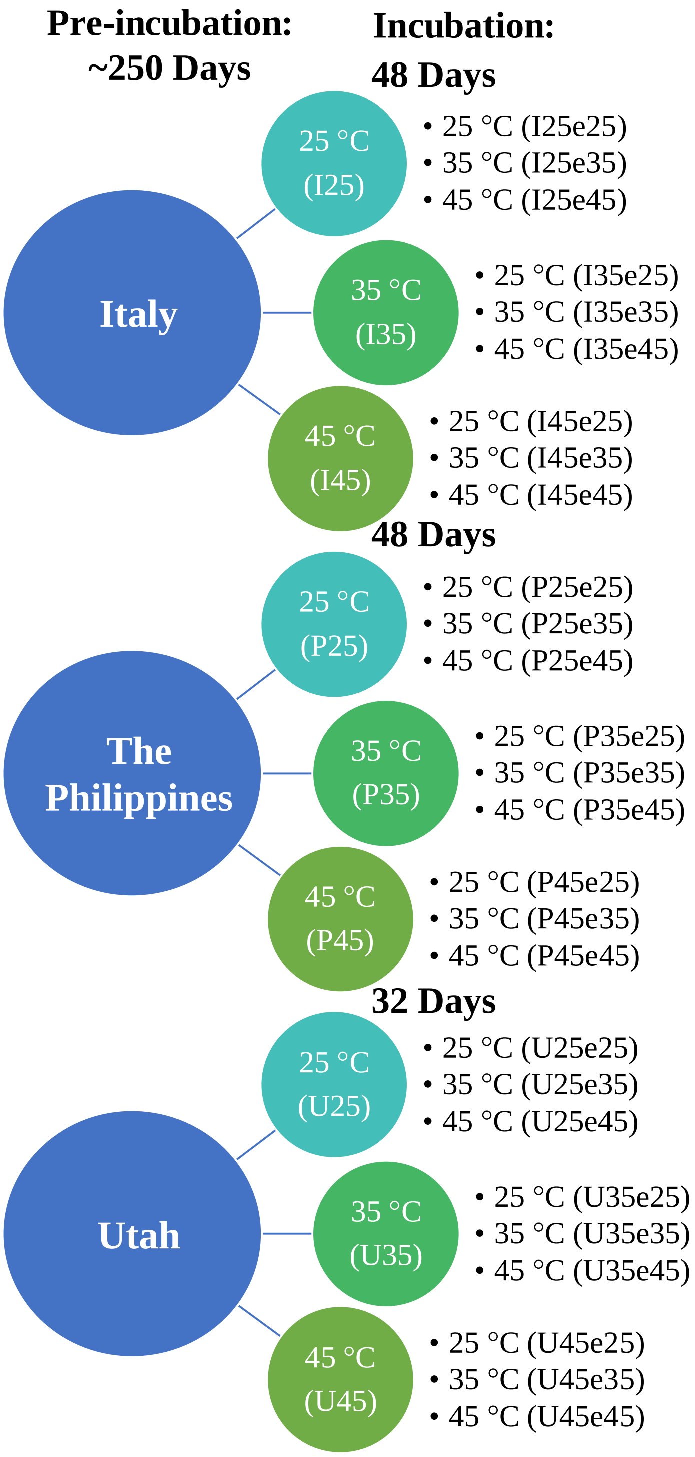

Figure S1. Experiment setup outline. Anoxic soil incubations were separated into two phases. For detailed descriptions, please refer to Materials and Methods in the main text. Time for the experiments setup were as following: Italian paddy soil and Philippines paddy soil: the pre-incubation were set up in Feb, 2015 and the incubation were set up in Oct, 2015;
Utah soil: the pre-incubation were set up in May, 2015 and the incubation were set up in Jan, 2016.

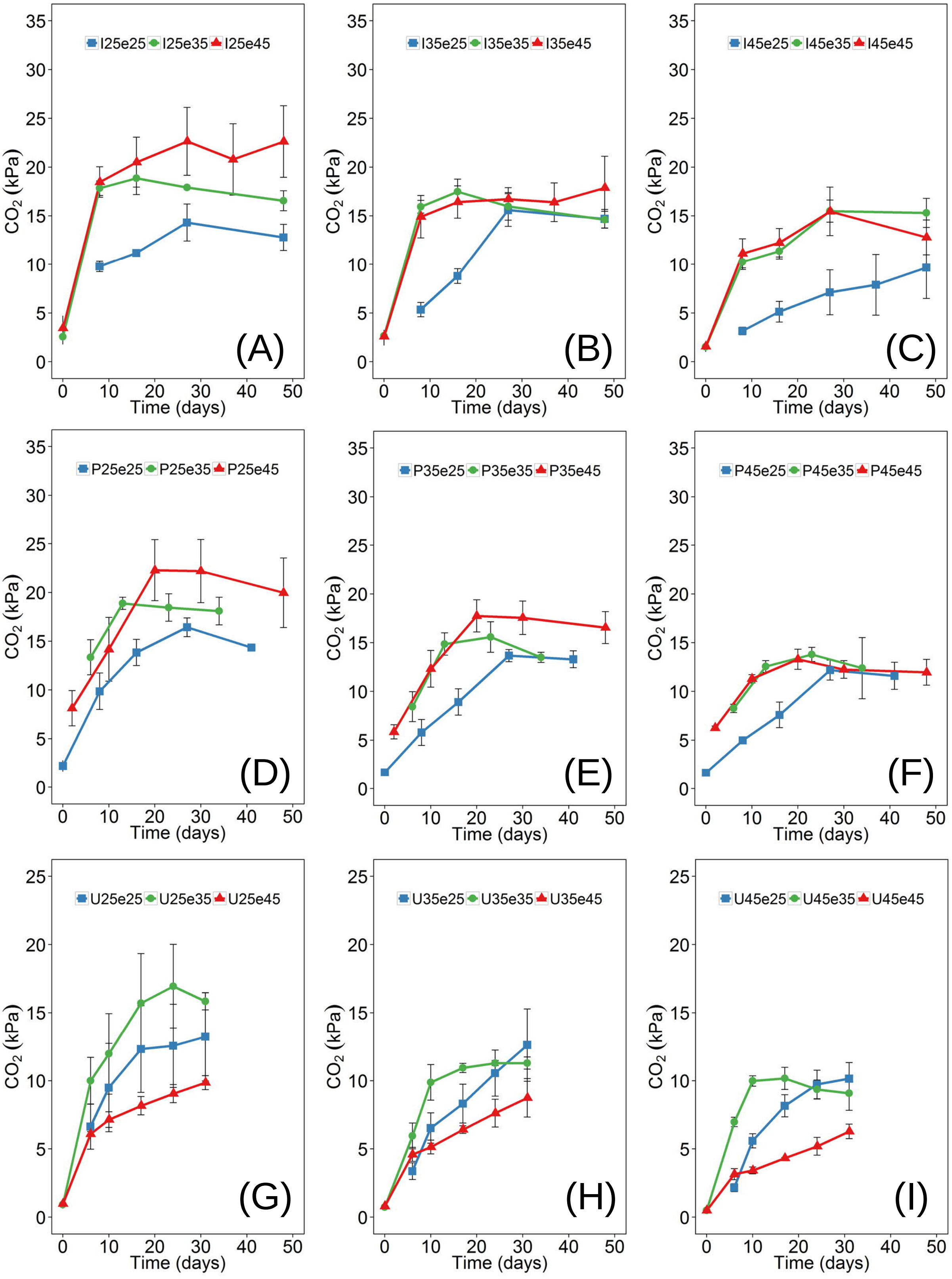

Figure S2. Time courses of the production of CO_2 in Italian (A-C), the Philippines (D-F) paddy soil and Utah (G-I) desert soil at different temperatures. Detailed descriptions of each treatment are given in Materials and Methods. Data shown are means ± SD, with n = 3.

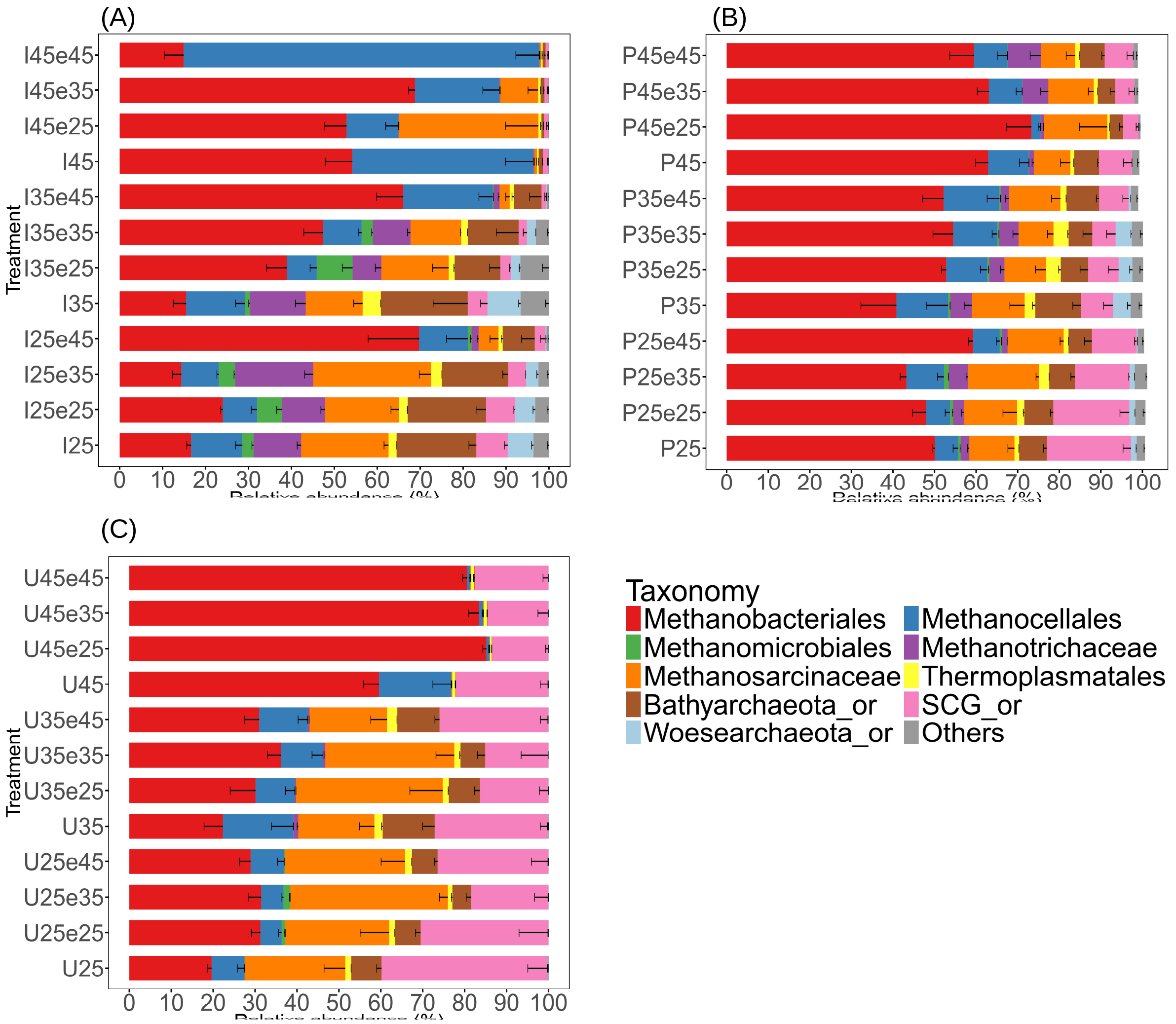

Figure S3. Composition of archaeal 16S rRNA gene sequences in (A) Italian paddy soil, (B) the Philippines paddy soil and (C) Utah desert soil; data are means ± SD (n = 3). Abbreviations: _or, order; SCG, Soil Crenarchaeota Group. See Materials and Methods in the main text for the description of each treatment. Data from the preincubation (I25, 35, 45, P25, 35, 45 and U25, 35, 45) were reported in our previous paper (Liu et al., 2018, Soil Biology and Biochemistry, 124,236-244), which are inlcuded here to show the start point community composition of each temperature treatment.

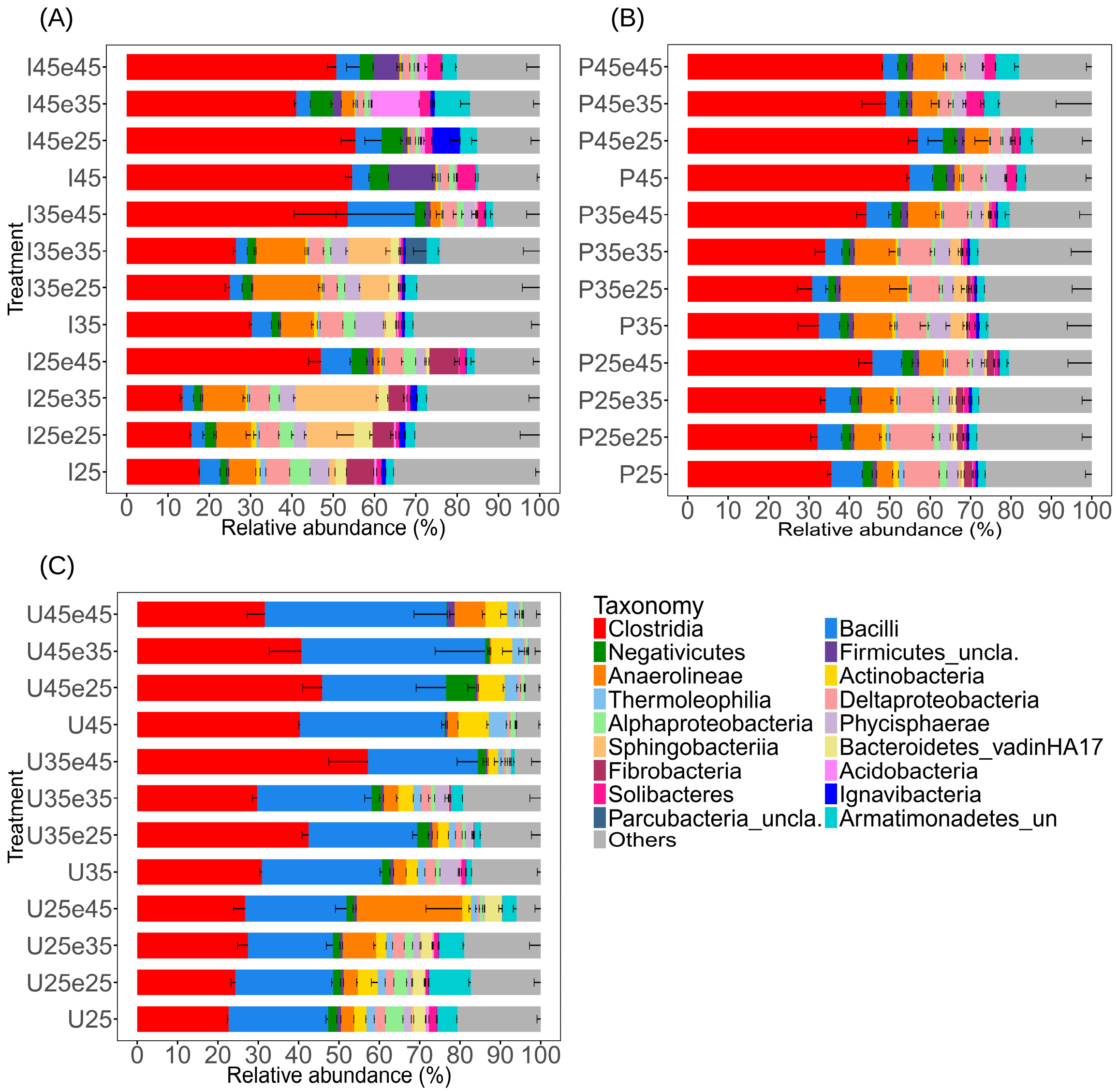

Figure S4. Composition of bacterial 16S rRNA gene sequences in (A) Italian paddy soil, (B) the Philippines paddy soil and (C) Utah desert soil. Data are shown at the taxonomic Class level. Only the Classes with maximum relative abundance > 2% (across all samples) are shown, and the remaining ones are combined as Others. Data are means ± SD (n = 3). Abbreviations: un, unclassified. See Materials and Methods in the main text for the description of each treatment. Data from the preincubation (I25, 35, 45, P25, 35, 45 and U25, 35, 45) were reported in our previous paper (Liu et al., 2018, Soil Biology and Biochemistry, 124,236-244), which are inlcuded here to show the start point community composition of each temperature treatment.

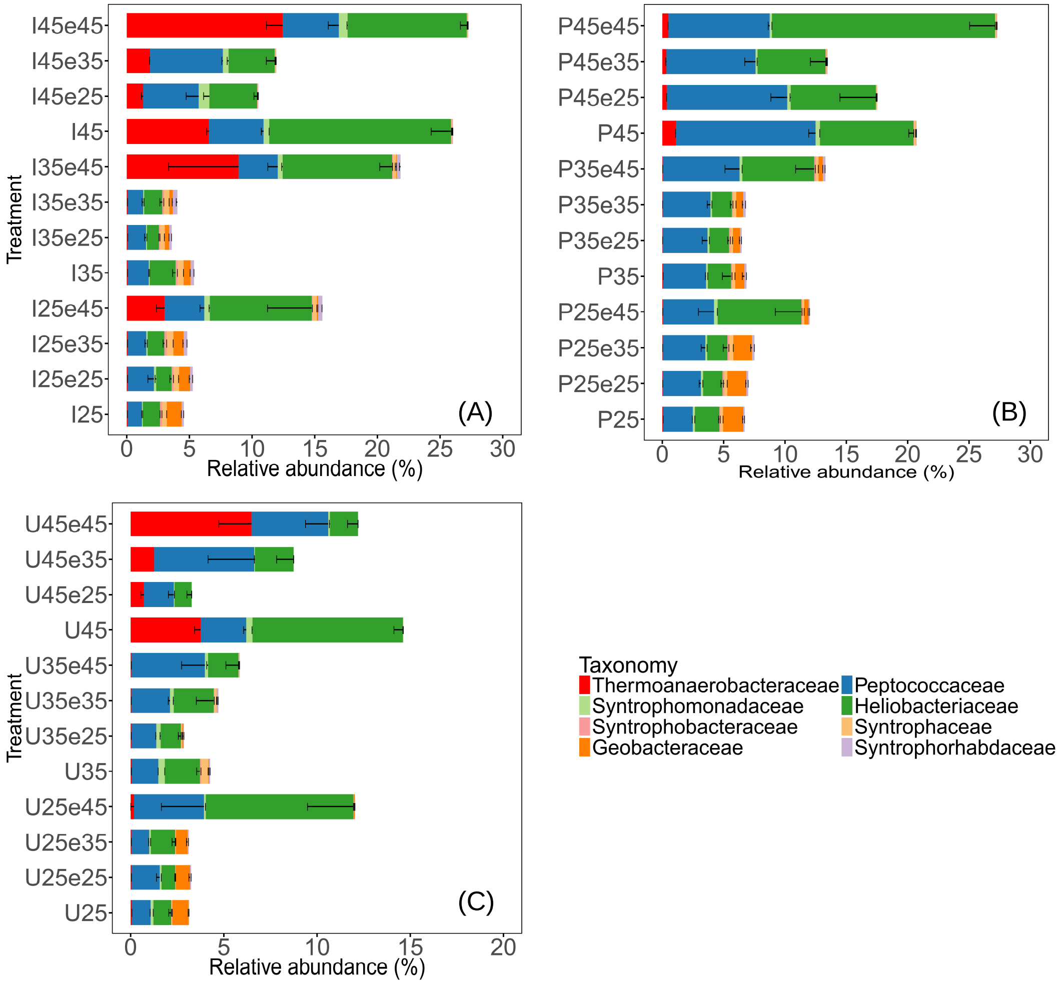

Figure S5. Relative abundances of putative syntrophic bacterial taxa (family level) in (A) Italian paddy soil, (B) the Philippines paddy soil and (C) Utah desert soil; data are means ± SD (n = 3). Note that the scale of the y axis is different in the three panels. See Materials and Methods in the main text for the description of each treatment. Data from the preincubation (I25, 35, 45, P25, 35, 45 and U25, 35, 45) were reported in our previous paper (Liu et al., 2018, Soil Biology and Biochemistry, 124,236-244), which are inlcuded here to show the start point community composition of each temperature treatment.

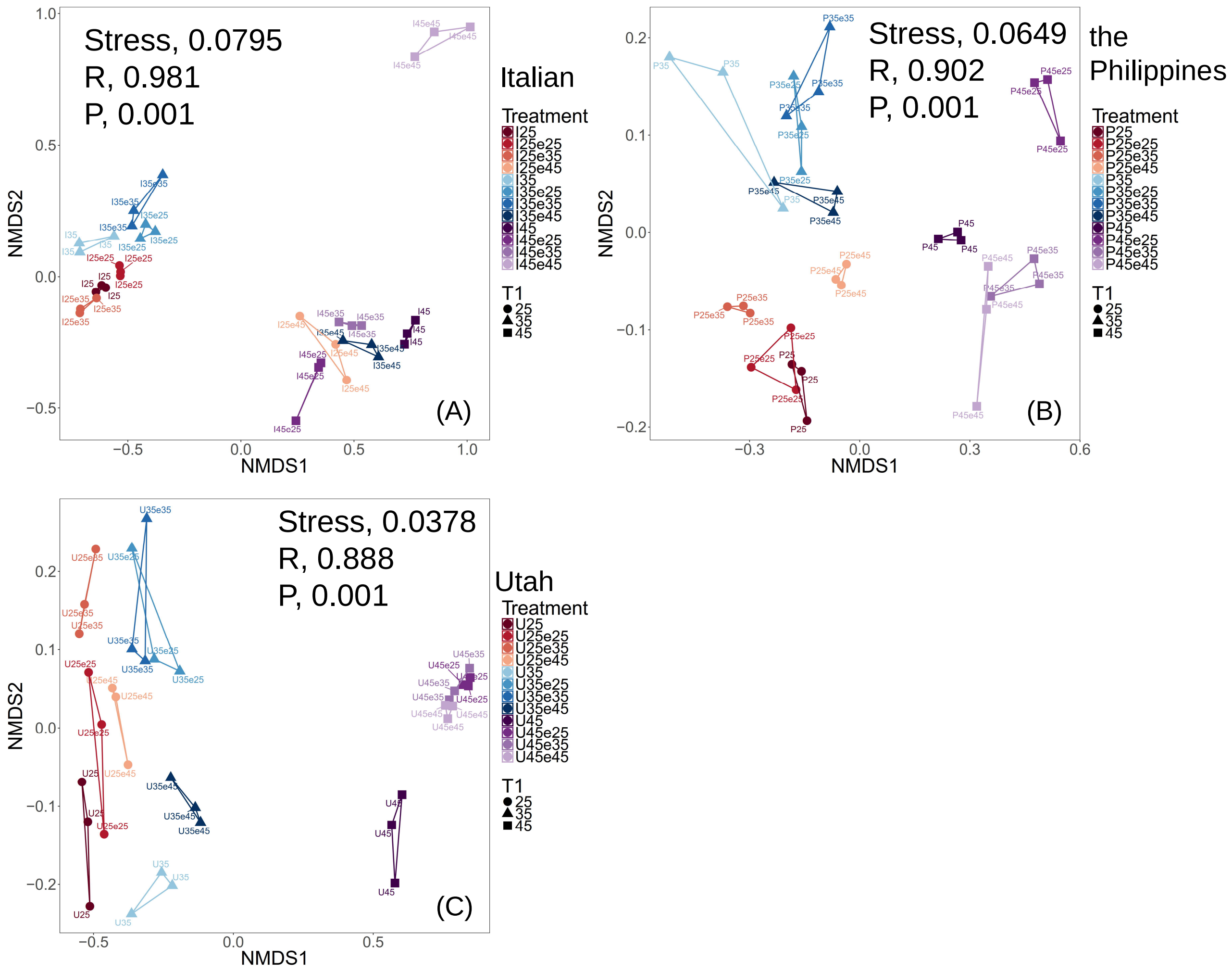

Figure S6. Non-metric multidimensional scaling (NMDS) analysis based on abundances of archaeal 16S rRNA gene OTUs (97% sequence similarity). (A), Italian, (B), the Philippines paddy soils and (C), Utah desert soil. Community similarities (Bray-Curtis) were calculated based on OTUs with a maximum relative abundance of ≥ 0.1% within each soil and 3 independent replicates were used for each treatment. T1 shown in the legend indicated the temperatures in pre-incubtion. The R- and P- values from the ANOSIM analysis are shown in the plot. Detailed descriptions of each treatment are given in Materials and Methods.

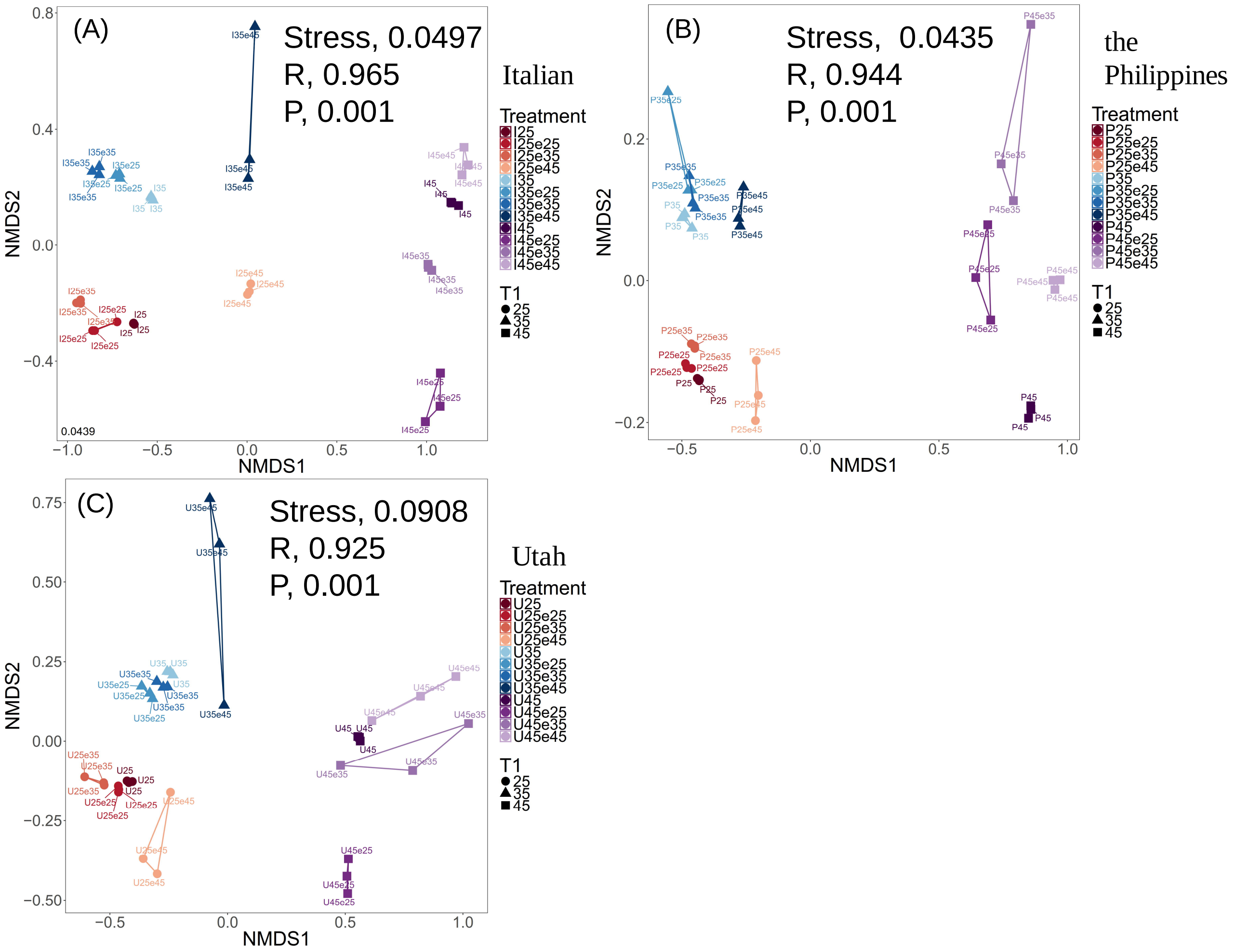

Figure S7. Non-metric multidimensional scaling (NMDS) analysis based on abundances of bacterial16S rRNA gene OTUs (97% sequence similarity). (A), Italian, (B), the Philippines paddy soils and (C), Utah desert soil. Community similarities (Bray-Curtis) were calculated based on OTUs with a maximum relative abundance of ≥ 0.1% within each soil and 3 independent replicates were used for each treatment. T1 shown in the legend indicated the temperatures in pre-incubation. The R- and P- values from the ANOSIM analysis are shown in the plot. Detailed descriptions of each treatment are given in Materials and Methods.

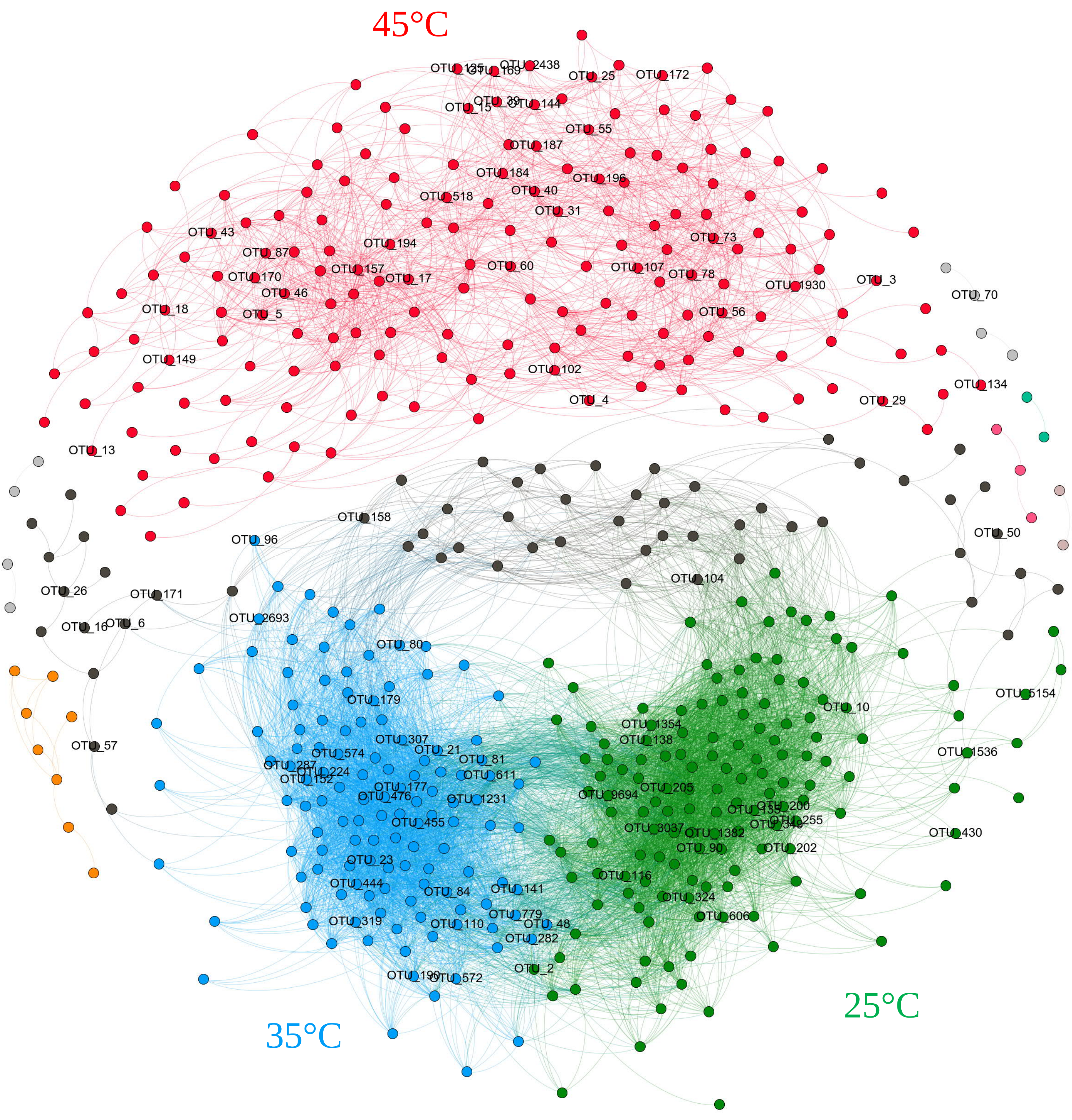

Figure S8. Network analysis of the co-occurrences of OTUs (included both Archaea and Bacteria) in Italian paddy soil. Nodes with OTU names are present in the heatmaps (see Fig.6A and Fig. 7 in the main text). Different modules are assigned by different colours. The modular name of the three major modules were set according to the clustering of OTUs in the heatmap. For example, OTUs in 45°C module generally showed highest relative abundances in the cluster with pre-incubation at 45°C.

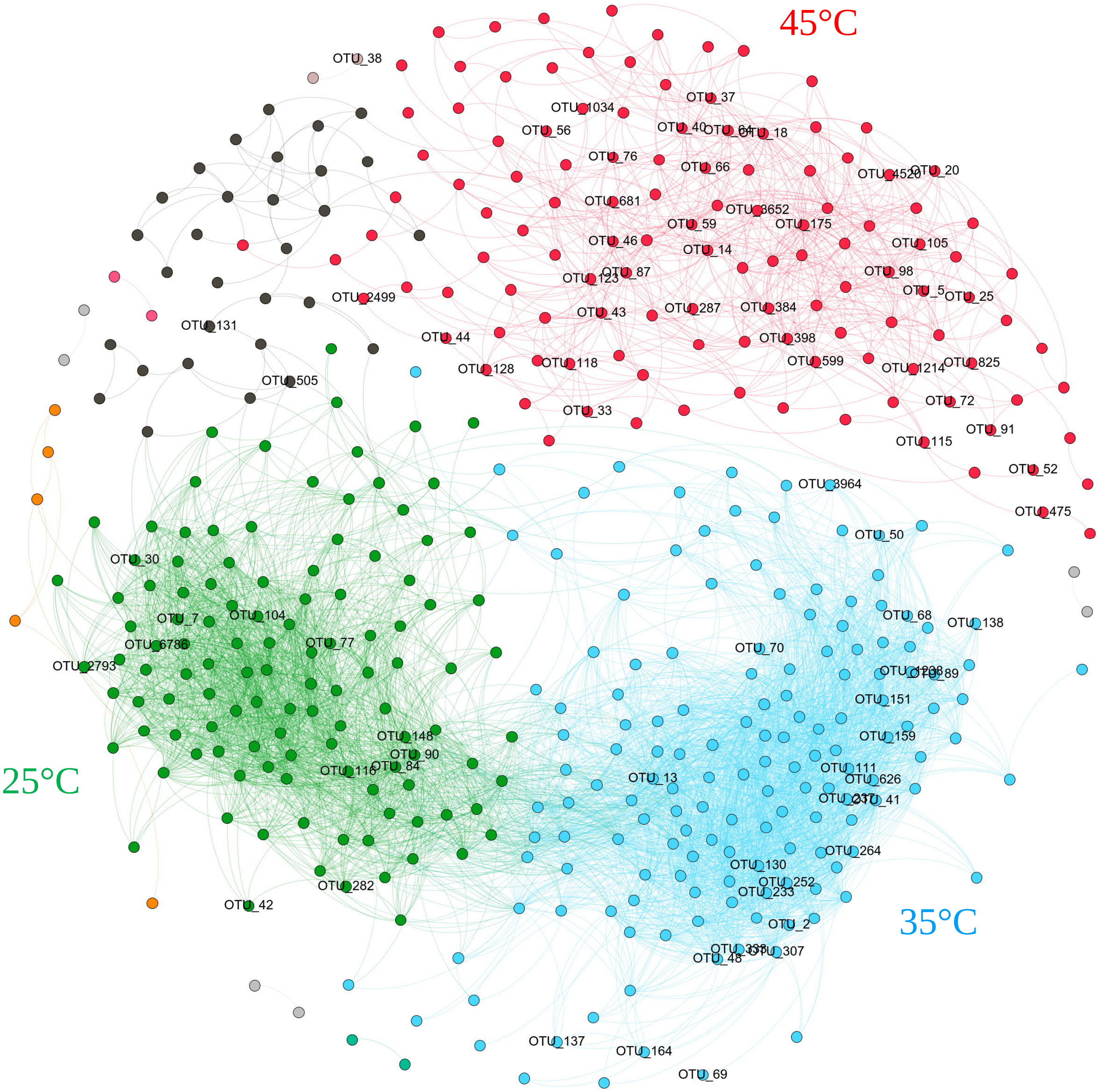

Figure S9. Network analysis of the co-occurrences of OTUs (included both Archaea and Bacteria) in the Phillipines paddy soil. Nodes with OTU names are present in the heatmaps (see Fig.6B and Fig. 8 in the main text). Different modules are assigned by different colours. The modular name of the three major modules were set according to the clustering of OTUs in the heatmap. For example, OTUs in 45°C module generally showed highest relative abundances in the cluster with pre-incubation at 45°C.

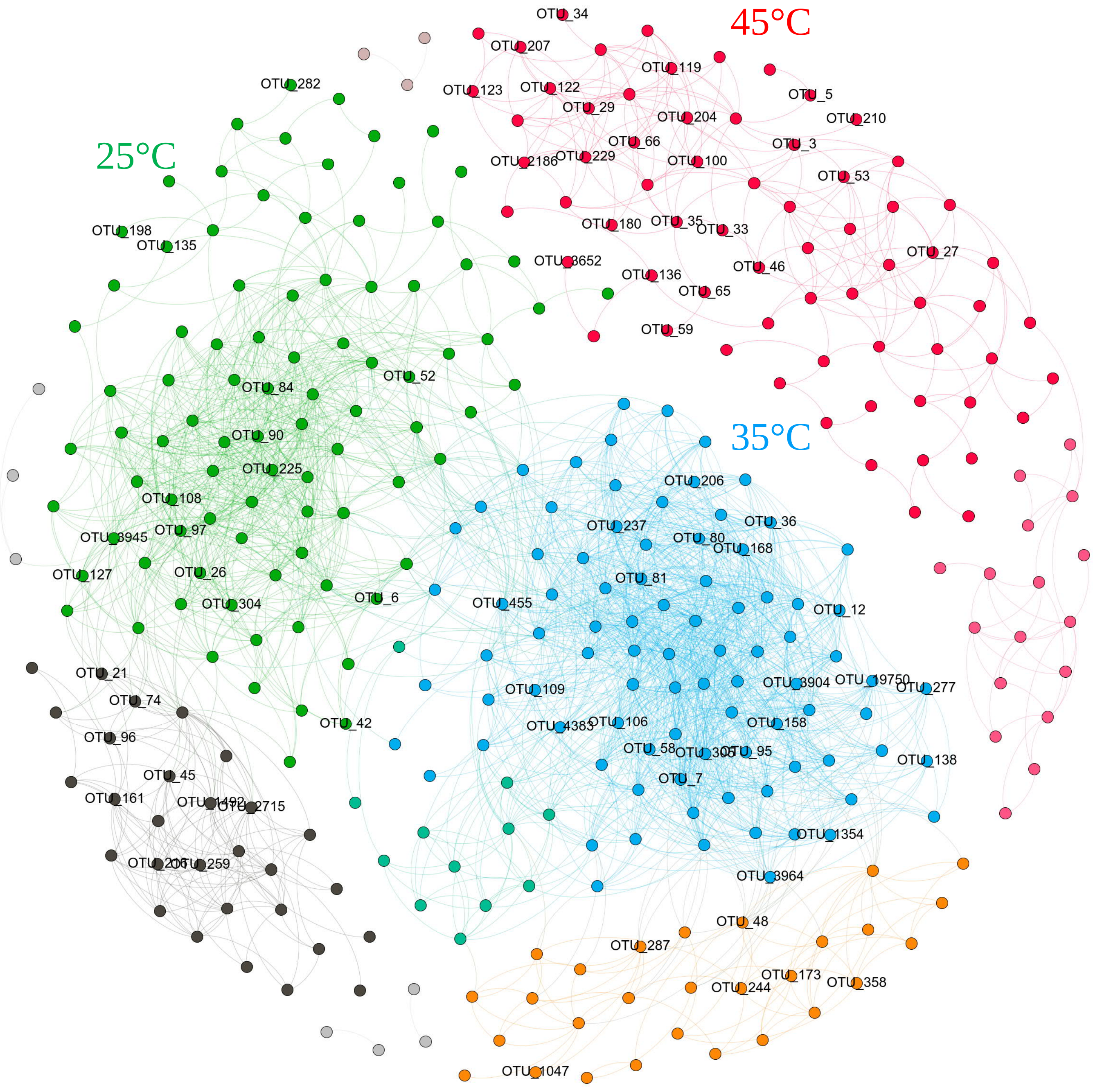

Figure S10. Network analysis of the co-occurrences of OTUs (included both Archaea and Bacteria) in Utah soil. Nodes with OTU names are present in the heatmaps (see Fig.6C and Fig. 9 in the main text). Different modules are assigned by different colours. The modular name of the three major modules were set according to the clustering of OTUs in the heatmap. For example, OTUs in 45°C module generally showed highest relative abundances in the cluster with pre-incubation at 45°C.
